# Cranial osteology and neuroanatomy of the late Permian reptile *Milleropsis pricei* and implications for early reptile evolution

**DOI:** 10.1098/rsos.241298

**Published:** 2025-01-08

**Authors:** Xavier A. Jenkins, Roger B. J. Benson, David P. Ford, Claire Browning, Vincent Fernandez, Elizabeth Griffiths, Jonah Choiniere, Brandon R. Peecook

**Affiliations:** ^1^ Department of Biological Sciences, Idaho State University, Pocatello, Idaho, USA; ^2^ Idaho Museum of Natural History, Pocatello, Idaho, USA; ^3^ American Museum of Natural History, New York NY, USA; ^4^ Evolutionary Studies Institute, University of the Witwatersrand, Johannesburg, South Africa; ^5^ Natural History Museum, South Kensington, London, UK; ^6^ Iziko Museums of South Africa, P.O. Box 61, Cape Town 8000, South Africa; ^7^ European Synchrotron Radiation Facility, 71 Avenue des Martyrs, Grenoble 38000, France; ^8^ Department of Earth Sciences, University of Oxford, Oxford, UK

**Keywords:** osteology, neuroanatomy, late Permian, Reptilia

## Abstract

Millerettidae are a group of superficially lizard-like Permian stem reptiles originally hypothesized as relevant to the ancestry of the reptile crown group, and particularly to lepidosaurs and archosaurs. Since the advent of cladistics, millerettids have typically been considered to be more distant relatives of crown reptiles as the earliest-diverging parareptiles and therefore outside of ‘Eureptilia’. Despite this cladistic consensus, some conspicuous features of millerettid anatomy invite reconsideration of their relationships. We provide a detailed description of the late Permian millerettid *Milleropsis pricei* using synchrotron X-ray phase-contrast micro-computed tomography focusing on the cranial anatomy of three individuals known from a burrow aggregation. Our data reveal a suite of neuroanatomical features *Milleropsis* shares with neodiapsids that are absent both in other ‘parareptiles’ and in early diverging groups of ‘eureptiles’. Traits shared between Milleropsis and neodiapsids include: the presence of a tympanic emargination on the quadrate, quadratojugal and squamosal, the loss of epipterygoid contribution to the basicranial articulation suggesting a more kinetic palatoquadrate, the absence of a sphenethmoid and the pathway of the abducens nerve through the braincase. Our findings suggest that the early reptile neurocranium, a region poorly sampled in phylogenetic analyses due to relative visual inaccessibility and poor preservation, has the potential to inform the phylogenetic relationships of early reptiles.

## 1. Introduction

Understanding the origins of modern reptiles has been confounded by patterns of available fossiliferous rock in which to sample terrestrial vertebrates in the late Palaeozoic [1,2], and an approximately 60-million-year-long period with several expansive ghost lineages between the earliest diapsid *Petrolacosaurus kansensis* [3,4], and the appearance of the reptile crown group Sauria under prevailing phylogenetic paradigms ([5–7], but see [8]). Modern cladistic analyses have reinforced the concept of extensive ghost lineages for the reptile crown stretching from the latest Carboniferous to the late Permian [5], organizing reptile clades into two groups, Eureptilia and Parareptilia [9].

Eureptilia, as proposed in previous studies (e.g. [10]), includes crown group reptiles plus their hypothesized close relatives, including the Permo-Carboniferous Captorhinidae and Araeoscelidia, and the late Permian non-saurian neodiapsids [5]. Parareptilia, as proposed [10], is more distantly related to the reptile crown group, and has typically been hypothesized to include the morphologically disparate clades such as the Permo-Carboniferous bolosaurids and ankyramorphans, the Early Permian mesosaurids, and the middle and late Permian millerettids [9,11].

The exclusively South African Millerettidae are generally considered to be the earliest diverging members of Parareptilia. This phylogenetic hypothesis introduces stratigraphic inconsistency because millerettids are the youngest ‘parareptile’ group to appear in the fossil record, in the latest Guadalupian (middle Permian [12–14]). More recent work, however, has challenged the existence of a eureptile–parareptile dichotomy, suggesting that ‘parareptiles’ may have a more crownward phylogenetic position than some ‘eureptile’ groups [15,16], and that Parareptilia may be paraphyletic, forming a grade of relatively crownward stem reptiles, with the Millerettidae placed as early diverging members of Neoreptilia [8] or as part of a smaller assemblage of former ‘parareptiles’ that excludes mesosaurids, bolosaurids and procolophonians [17].

Pre-cladistic studies of millerettids argued for their affinities closer to Sauria. The first described millerettid, *Broomia perplexa*, was interpreted as a reptile of uncertain affinities, although Watson [18] noted anatomical similarities with ‘early lizards’ including *Sphenodon* [18]. In the seminal reviews of reptile evolution by both Romer [19] and Watson [20], millerettids were placed as ancestral to, or within, *‘*Eosuchia’, a paraphyletic grade of stem reptiles approximately equivalent to Neodiapsida today that included small and gracile diapsid taxa from the Permo-Triassic [19,21]. Other works on early reptile evolution proposed that the millerettids were within Lepidosauria [22,23] or Archosauria [24], whereas more recent studies suggested that the millerettids gave rise to an assemblage of stem lepidosaurs similar to ‘Lepidosauromorpha’ of today and even including the early archosauromorph *Prolacerta* (thought to be a lepidosaur at the time) based on aspects of the palatoquadrate and tympanic fossa and similarities between *Milleretta* and ‘eosuchians’ [25].

Despite this early work noting similarities between millerettids and crown reptiles, millerettids have been placed into Parareptilia [9] since the advent of cladistics (but see [8]). The placement of millerettids in these studies was based primarily on superficially ‘primitive’ characters present in *Milleretta* [5,11,26], although these studies also have often noted that some traits of millerettids differ from the anatomy of other ‘parareptiles’. These phylogenetic classification schemes, both before and after cladistic methods, were highly dependent on patterns of temporal fenestration [15,23,27,28]. When *Milleretta* was described by Broom [29] (under the occupied name ‘*Millerina*’) and later by Broom [30], it was noted as having superficial similarities to ‘cotylosaurs’ such as *Captorhinus*, emphasizing gestalt similarities of the skull roof including the anapsid skull of the holotype specimen. However, later discoveries demonstrated that young individuals of *Milleretta* have an open lower temporal fenestra, which closes through ontogeny [25]. Notably, *Milleretta* is the only known millerettid in which this occurs; all other known millerettids possess a lower temporal emargination throughout ontogeny [12,20,25]. This reflects a broader principle in phylogenetic inference, that early and anatomically primitive members of groups may be more informative about phylogenetic relationship and also have greater relevance to evolutionary change on the reptile stem lineage [31,32]. The apparent conflict between the phylogenetic position of Milleretidae implied by historical studies and that put forward by more recent cladistic work invites reconsideration of the anatomical evidence for the affinities of the group. A more complete understanding of the plesiomorphic millerettid *Milleropsis pricei* [20,25] is therefore necessary to infer both the internal relationships of the Millerettidae and their phylogenetic placement among other stem reptiles.

Here, we use propagation phase-contrast synchrotron X-ray phase-contrast micro-computed tomography (PPC-SRµCT) to produce three-dimensional digital reconstructions of the cranial anatomy of the holotype of the plesiomorphic millerettid *Milleropsis*, BP/1/720, a specimen of at least nine semi-articulated individuals preserved in a burrow aggregation [20]. These reconstructions reveal remarkable detail of the cranial osteology and neurocranium of this taxon, including aspects of the basicranial articulation, the pathway of the cranial nerves and carotid arteries through the braincase, and permit digital reconstruction of the endosseous labyrinth. The reconstructions reveal anatomical features that *Milleropsis*, and other millerettids, share with Sauria, suggesting more crownward affinities for millerettids than in current phylogenetic paradigms.

## 2. Material and methods

**Figure 1.**
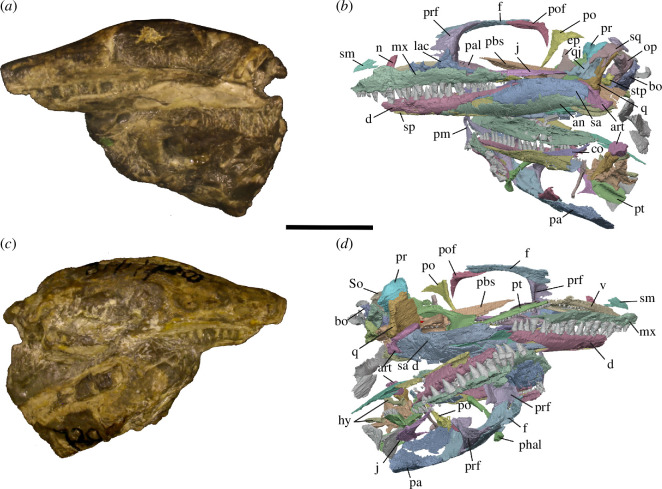
BP/1/720, holotype of *Milleropsis pricei*. (*a*) Left lateral view; (*b*) left lateral view of segmented elements from µCT scan of the specimen; (*c*) right lateral view; (*d*) right lateral view of segmented elements from µCT scan of the specimen. Abbreviations: an, angular; art; articular; bo, basioccipital; co, coronoid; d, dentary; ect, ectopterygoid; epi, epipterygoid; fr, frontal; hy, hyoid; j, jugal; lac, lacrimal; mx, maxilla; n, nasal; op, opisthotic; pa, parietal; pal, palatine; pbs, parabasisphenoid; phal, phalanges; pm, premaxilla; po, postorbital; pof, postfrontal; pr, prootic; pra; prearticular; prf, prefrontal; pt, pterygoid; q, quadrate; qj, quadratojugal; sa, surangular; sm, septomaxilla; so, supraoccipital; sp, splenial; sq, squamosal; stp, stapes; and v, vomer. Scale bar represents 1 cm.

The holotype skull of *Milleropsis pricei* (BP/1/720, figure 1) was characterized at the ID19 beamline of the European Synchrotron Radiation Facility (ESRF, Grenoble, France) using PPC-SRµCT. The set-up consisted of: filtered white beam (W150B-67.50 mm gap; Cu 5 mm; total integrated detected energy 91.1 keV); 5.6 m propagation distance; indirect detector (GGG 1000 µm scintillator, 0.67× magnification from two Hasselblad photo lenses, PCO.edge 4.2 sCMOS camera); resulting measured isotropic pixel size of 8.86 µm.

To compensate for the limited field of view of 1088 × 2048 pixels (vertical × horizontal): the centre of rotation was shifted by 7.088 mm (i.e. 800 pixels), allowing reconstruction of 36 482 pixel slices; four acquisitions were done, moving the specimen on the vertical axis by 8.435 mm in between scans (12% overlap between acquisitions). Each acquisition consisted of 5000 projections, with 30 ms exposure time per frame (no accumulation). Reference images for flatfield (*n* = 41) and dark noise current (*n* = 40) correction were only recorded once for the whole series of acquisition. Prior to the tomographic reconstruction, radiographs were stitched vertically following the protocol described in Benoit *et al*. [33]. Tomographic reconstruction was done using the single distance phase retrieval approach of PyHST2 [34,35]. Normalization of artefactual grey level gradients and metallic inclusions was performed on the resulting 32-bit image stack [36]. Remaining post processing included: change of dynamic range from 32-bit to 16-bit; ring correction [37]; cropping; binning 2 × 2 × 2, resulting in a final dataset with isotropic voxel size of 17.72 µm (Matlab code available at https://github.com/HiPCTProject/Tomo_Recon). To facilitate segmentation, the dynamic range was further rescaled to 8-bit. The processed dataset (with a resulting isometric voxel size of 17.72 µm) is available on Morphosource (https://www.morphosource.org/concern/media/000125334).

**Figure 2.**
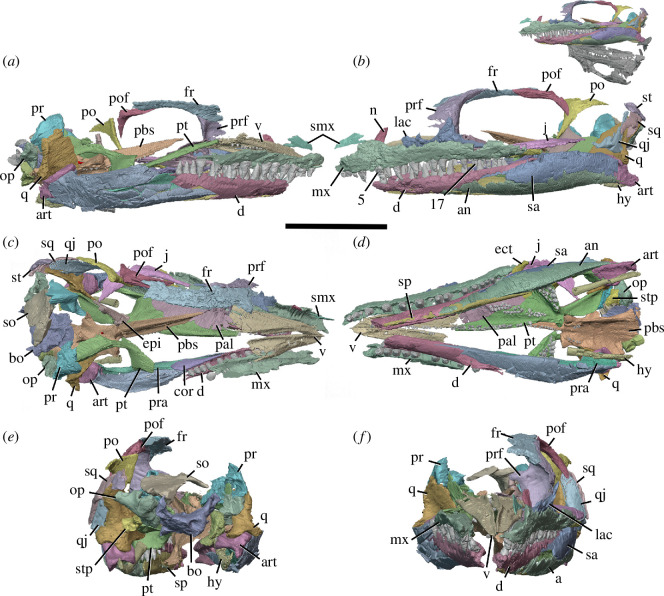
BP/1/720, holotype of *Milleropsis pricei*. Segmented elements from µCT scan of Individual II in: (*a*) left lateral; (*b*) right lateral; (*c*) dorsal; (*d*) ventral; (*e*) occipital and (*f*) anterior views. Abbreviations: an, angular; art; articular; bo, basioccipital; cor, coronoid; d, dentary; ect, ectopterygoid; epi, epipterygoid; f, frontal; hy, hyoid; j, jugal; lac, lacrimal; mx, maxilla; n, nasal; op, opisthotic; pal, palatine; pbs, parabasisphenoid; po, postorbital; pof, postfrontal; pra; prearticular; prf, prefrontal; pr, prootic; pt, pterygoid; q, quadrate; qj, quadratojugal; sa, surangular; smx, septomaxilla; sp, splenial; sq, squamosal; st, supratemporal; stp, stapes; v, vomer; 5, fifth maxillary tooth position; and 17, 17th maxillary tooth position. Scale bar represents 1 cm.

**Figure 3.**
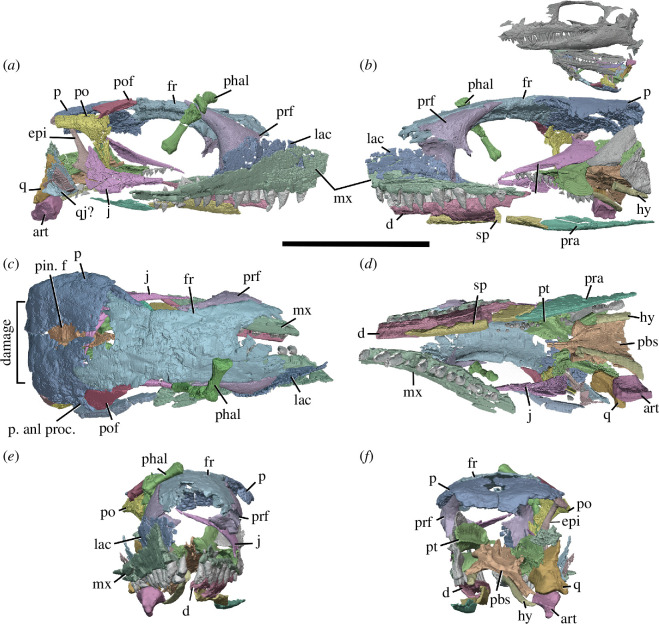
BP/1/720, holotype of *Milleropsis pricei*. Segmented elements from µCT scan of Individual VI in: (*a*) left lateral; (*b*) right lateral; (*c*) dorsal; (*d*) ventral; (*e*) occipital and (*f*) anterior views. Abbreviations: an, angular; art; articular; cor, coronoid; d, dentary; ect, ectopterygoid; epi, epipterygoid; hy, hyoid; j, jugal; lac, lacrimal; mx, maxilla; n, nasal; op, opisthotic; p, parietal; p. anl proc, anterolateral process of parietal; pbs, parabasisphenoid; phal, phalanges; po, postorbital; pof, postfrontal; pr, prootic; pra; prearticular; prf, prefrontal; pt, pterygoid; q, quadrate; sa, surangular; smx, septomaxilla; sp, splenial; and stp, stapes. Scale bar represents 1 cm.

**Figure 4.**
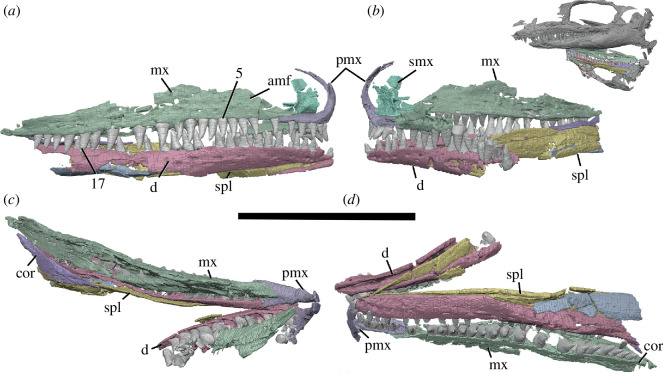
BP/1/720, holotype of *Milleropsis pricei*. Segmented elements from µCT scan of Individual IX in: (*a*) left lateral; (*b*) right lateral; (*c*) dorsal and (*d*) ventral views. Abbreviations: amf, anterior maxillary foramen; cor, coronoid; d, dentary; mx, maxilla; pmx, premaxilla; smx, septomaxilla; spl, splenial; 5, fifth maxillary tooth position; and 17, 17th maxillary tooth position. Scale bar represents 1 cm.

Watson [20] identified multiple individuals in BP/1/720, using roman numerals I–X and a corresponding colour (painted on each individual) to identify each one. He designated the largest individual (II) as the holotype (figure 2). We scanned the nodule containing the skull of the holotype, which contained the skulls of individual VI (figure 3) and an additional individual IX (figure 4). These skulls are approximately 60% and 65% the length of the holotype individual II. We interpret these smaller individuals as immature because they have a more weakly ossified braincase and otic capsule.

All specimens were digitally segmented using Dragonfly version 2022.2 for Windows (https://www.theobjects.com/dragonfly). High-resolution regions of interest highlighting aspects of anatomy were exported as .stl models and then imported into Blender 3.10 (https://www.blender.org), where they were rendered as two-dimensional images to be used in figures for this study. Skull reconstructions were created in Blender using composites of the three individuals present in the tomography data. The holotype, Individual II, was used as a base, and elements from Individual VI (upscaled by 40%) and Individual IX (upscaled by 35%) were added to the reconstruction. Duplication and mirror imaging were used to replace missing or damaged elements.

## 3. Systematic palaeontology

AMNIOTA Haeckel, 1866

SAUROPSIDA Huxley, 1864

NEOREPTILIA Ford & Benson, 2020

Millerettidae Camp *et al*., 1949

Genus *Milleropsis* Gow, 1972

Type species: *Millerosaurus pricei* Watson, 1957

*Milleropsis pricei* [20]

### 3.1. Material studied

BP/1/720 (holotype), a nodule containing at least nine partially articulated individuals based primarily on the number of exposed mandibles and maxillae, although many of these specimens have been separated from the main nodule by mechanical preparation [20]. Individuals VII and VIII could not be relocated with certainty, in part due absence of the original paint used by Watson [20] to identify each individual. BP/1/4203 (referred specimen), nearly complete, articulated skeleton. SAM-PK-K7751, a nearly complete mediolaterally crushed skull with well-developed cranial ‘osteoderms’. SAM-PK-K10082, an almost complete dorsoventrally crushed skull with a damaged temporal region but with a well-preserved antorbital region. SAM-PK-K8609, a mostly complete, uncrushed skull exhibiting well-preserved dermal sculpting.

### 3.2. Locality and horizon

Swaelkrans, Murraysburg, South Africa; Middle *Cistecephalus* Assemblage Zone (BP/1/720). Nieu Bethesda, Graaff-Reinet, South Africa; Middle *Cistecephalus* Assemblage Zone (BP/1/4203). Kafferskraal 42, Beaufort West, South Africa; *Endothiodon* Assemblage Zone, *Tropidostoma-Gorgonops* Subzone (SAM-PK-K7751). Leeukloof 43, Beaufort West, South Africa; *Endothiodon* Assemblage Zone, *Tropidostoma-Gorgonops* Subzone (SAM-PK-K8609). Quaggafontein 83, Beaufort West, South Africa; *Endothiodon* Assemblage Zone, *Tropidostoma-Gorgonops* Subzone (SAM-PK-K10082).

### 3.3. Revised diagnosis

*Milleropsis pricei* is a small-bodied (basal skull length approx. 3 cm) millerettid with the following unique combination of characters: the presence of dermal ornamentation present as low bosses that do not extend onto the jugal or prefrontal*, five premaxillary teeth, 18 or more maxillary teeth*, a moderately expanded external naris, an elongated nasal similar in length to the frontal*, a short lacrimal that does not contribute to the external naris, a parietal bearing a ventrolateral flange, a posterior spur-like process of the jugal entering a lower temporal emargination*, a dorsal process of the quadratojugal visible in medial view, a tympanic emargination between the squamosal, quadratojugal and quadrate, two enlarged vomerine teeth anteriorly on the vomer, a transversely narrow parabasiphenoid body, a prootic that bears a notch instead of a foramen for CN VII, a small stapes without a dorsal process, a distally expanded stapedial shaft, 20 dorsal vertebrae*, unexpanded ribs*, two pairs of lateral gastralia* and fused distal tarsals IV and V*. Asterisks represent autapomorphies that distinguish *Milleropsis* from other millerettids based on comparisons primarily with *Broomia perplexa* and *Milleretta rubidgei*.

## 4. Description

### 4.1. The skull

**Figure 5.**
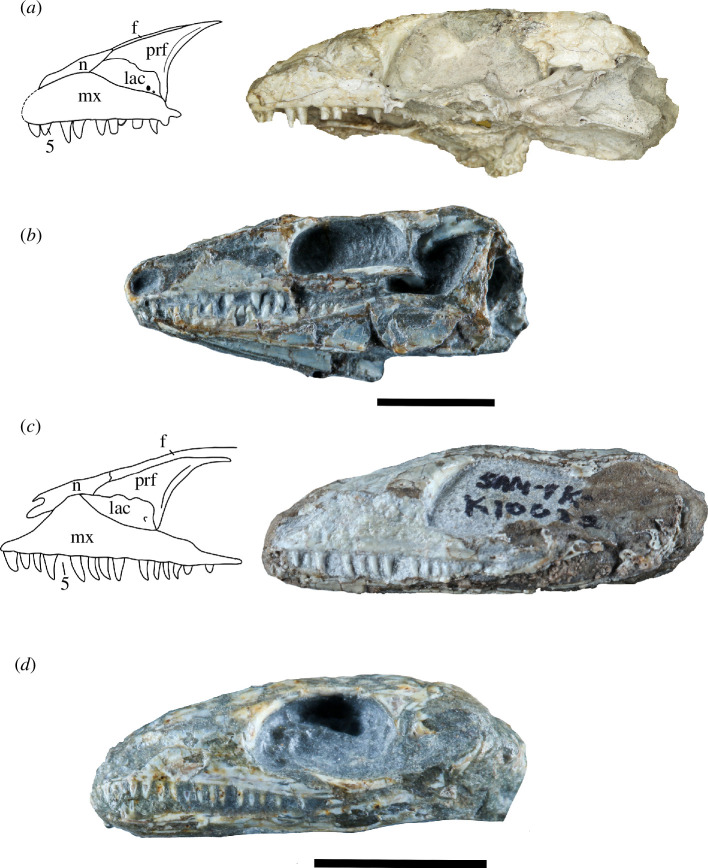
Specimens of *Milleropsis* not scanned in this study. (*a*) BP/1/720, holotype of *Milleropsis pricei*, Individual I in left lateral view with a corresponding line drawing demonstrating antorbital sutures; (*b*) SAM PK-K7751, referred specimen, left lateral view; (*c*) SAM-PK-K10082, referred specimen, left lateral view with corresponding line drawing; (*d*) SAM-PK-K8609, referred specimen, mirrored right lateral view. Scale bars each represent 1 cm.

The holotype of *Milleropsis pricei, ‘*Individual II’ of the multi-individual specimen BP/1/720 [20], was originally part of a nodule containing at least nine individuals. This nodule was subsequently prepared down to isolate two of the most complete skulls (Individual II and VI [20]). During this preparation, much of the exposed skull surfaces were damaged, in some cases altering the apparent sutural morphology, particularly in the antorbital region (figure 1). The following description is based on our digital reconstruction from synchrotron imaging of the block that includes Individual II, a nearly complete skull missing most of its skull roof due to historical preparation damage (figure 2). Individual II is slightly crushed and flattened, and the elements of the left circumorbital and temporal regions are disarticulated, causing the orbit to appear artificially larger. This block also includes two other, partial skulls: Individual VI of [20], comprising most of the posterior skull (figure 3), and another partial skull that we refer to as Individual IX, which was not recognized by Watson [20]. Individual IX preserves the mandibular symphysis (not preserved in Individual II) and the only premaxillae known in *Milleropsis*, demonstrating the anterodorsally expanded external naris of this taxon (figure 4). Our descriptions of individuals II, VI and IX are supplemented by the information from the other specimens described by Watson [20] and Gow [25] including Individual I (figure 5*a*), which was acid-prepared by Gow [25] and displays important information on the sutural relationships of the snout that was not well preserved in the individuals we scanned.

**Figure 6.**
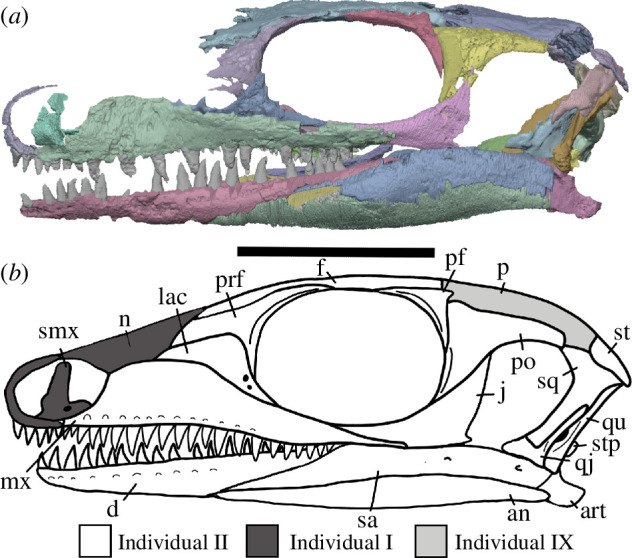
Reconstructions of *Milleropsis pricei* BP/1/720 in left lateral view. (*a*) Segmentation and (*b*) line drawing. Refer to key for shading. Abbreviations: an, angular; art; articular; d, dentary; j, jugal; lac, lacrimal; mx, maxilla; n, nasal; p, parietal; pm, premaxilla; pf, postfrontal; po, postorbital; prf, prefrontal; qu, quadrate; qj, quadratojugal; sa, surangular; smx, septomaxilla; st, supratemporal; stp, stapes; and sq, squamosal. Scale bar represents 1 cm.

Our descriptions are further supplemented by referred specimens SAM-PK-K7751, SAM-PK-K-8609 and SAM-PK-K10082 (figure 5*a–c*), which we refer to *Milleropsis* based on the following features: the presence of cranial osteoderms that are restricted to the skull roof, the presence of 18 or more maxillary teeth, a lacrimal that is excluded from the external naris via maxilla-nasal contact; a ventrolateral flange of the parietal, a large pineal foramen, the absence of an upper temporal fenestra and the presence of a lower temporal emargination, all features that distinguish *Milleropsis* from other millerettids as well as neodiapsids (Jenkins *et al*., [38] in review). BP/1/4203 was previously referred to *Milleropsis* by Gow [25] based on similarities between the manus of this specimen and BP/1/720, as well as the presence of characteristic cranial ornamentation on the frontals and parietals. Tomography data of BP/1/4203 (personal observation) reveal the presence of enlarged vomerine teeth, rows of teeth on the ventral plate of the parabasisphenoid, an open lower temporal emargination and a midline gastralial element. These observations support a millerettid, and specifically *Milleropsis*, referral of the material. Our reconstruction of the skull of *Milleropsis* (figure 6) therefore uses information from all the specimens available for study (BP/1/720, BP/1/4203, SAM-PK-K7751, SAM-PK-K-8609 and SAM-PK-K10082).

#### 4.1.1. Premaxilla

**Figure 7.**
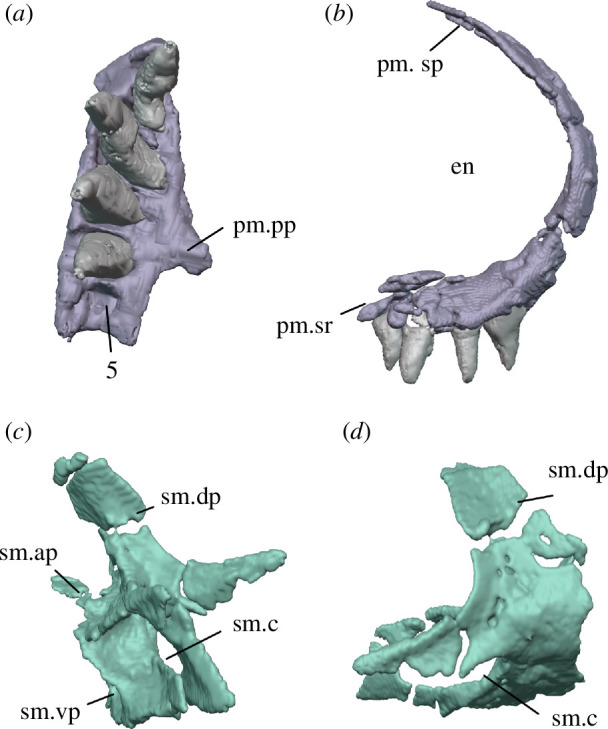
Right premaxilla and septomaxilla of *Milleropsis pricei* of BP/1/720, Individual IX. Premaxilla in ventral (*a*) and right lateral (*b*) views; right septomaxilla in posterior (*c*) and right lateral (*d*) views. Abbreviations: en, external naris; pm.pp, palatal process of premaxilla; pm.sp, supranarial process of premaxilla; pm.sr, maxillary process of premaxilla; sm.ap, anterior process of septomaxilla; sm.c, septomaxillary canal; sm.dp, dorsal process of septomaxilla; and sm.vp, ventral plate of septomaxilla.

The premaxillae of *Milleropsis pricei* have not previously been described because they were missing from all eight skulls described by Watson [20] and Gow [25]. The scans of BP/1/720 reveal the presence of two nearly complete premaxillae in articulation in Individual IX (figure 4). The left premaxilla is the more complete of the two and comprises a supranarial process anteriorly, a palatal process medially and a subnarial ramus posteriorly (figure 7*a,b*). The premaxilla of *Milleropsis* contributes to the anterodorsal margins of an enlarged external naris that is almost half the dorsoventral height of the orbit, similar to that of *Eunotosaurus africanus* [39]. The premaxilla contacts the maxilla laterally, the vomer and septomaxilla medially and the nasal posterodorsally.

The subnarial ramus of the premaxilla forms most of the ventral margin of the enlarged external naris (figure 4*a*). The anterior end of the dorsal surface of the subnarial ramus forms a sharp angle with the lateral surface, lacking the narial shelf that is widespread among early amniotes [40]. The lateral surface of the premaxilla of *Milleropsis* is smooth, lacking the dermal sculpting present in acleistorhinid stem reptiles such as *Acleistorhinus pteroticus* [41] and also the foramina piercing the lateral surface in *Petrolacosaurus* [3]. The subnarial process of the premaxilla is anteroposteriorly short, where it underlies and fits onto a ventrally facing notch on the maxilla, forming a short ‘tongue-and-groove joint’ (figure 4*d*, *sensu* [42]). A distinct maxillary process is absent, as in *Captorhinus laticeps* [43] and *Youngina capensis* (BP/1/2871), and differing from the distinct maxillary process in some other taxa, including *Petrolacosaurus* [3] and *Orovenator* ([15]; figure 4*a*).

The supranarial process of *Milleropsis* terminates just posterior to the midlength of the external naris, contributing to its anterior margin (figure 4*b*). The supranarial process of the premaxilla angles anterodorsally at about 15° from the vertical, forming a small ‘rostral process’ that extends beyond the anteriormost point of the tooth row. The supranarial process then inflects posterodorsally at about its midheight (figure 4). The weakly recumbent supranarial process of *Milleropsis* is similar to those of some varanodontines (e.g. *Aerosaurus* [44]), *Orovenator mayorum* [15] and *Eunotosaurus africanus* [45,46]. These differ from the strongly recumbent rostral process characteristic of recumbirostrans (e.g. *Quasicaecillia texana* [47]), the diadectomorph *Limnoscelis* [48], caseasaurs (e.g. *Eothyris parkeyi* [49] *Ennatosaurus tecton* [50]) and some procolophonids (e.g. *Leptopleuron lacertinum* [51]), which often slope anterodorsally at angles more than 60° from vertical The supranarial process contacts the nasal with a transversely flat suture (figure 4), unlike the double-pronged ‘alary process’ of captorhinids and some recumbirostrans, such as *Euconcordia cunninghami* [52,53] or *Huskerpeton englehorni* [54]. Termination of the supranarial process of *Milleropsis* just posterior to the midlength of the external naris is similar to the condition seen in procolophonians [55] and the neodiapsids *Youngina* [56] and *Claudiosaurus* [57]. This differs from most other early amniotes including: captorhinids [58], most synapsids [59] and non-archosauromorph reptiles [60] in which the supranarial process terminates at the anterodorsal margin of the external naris. It is also unlike the condition in bolosaurids (e.g. *Belebey vegrandis* [61]), araeoscelidians (e.g. *Araeoscelis* [62]) and *Orovenator* [15] in which the posterior end of the supranarial process is level with or posterior to the posterior margin of the external naris.

The palatal process of the premaxilla extends posteromedially at the level of the third alveolus, and terminates distally at the level of the fourth alveolus. The medial margin of the palatal process appears to be weakly emarginated, although this emargination probably did not receive an anteromedial process of the vomer as it lacks a distinct facet for this contact (figure 7). Posterior to this, the palatal process of the premaxilla bears a palatal process that projects posteromedially. The vomerine process is extremely reduced, not extending as far posteriorly as the maxillary process, as in most early amniotes including the stem-amniote *Seymouria* [63], the sphenacodontian *Dimetrodon milleri*, the araeoscelidian *Petrolacosaurus* [3] and neodiapsids [57]. This differs from the condition in procolophonians [64], in which the palatal process of the premaxilla contributes broadly to the palatal surface and extends posteriorly farther than the maxillary process. The choana, or internal naris, of *Milleropsis,* incises the palatal process of the premaxilla at the level of the vomerine process.

**Figure 8.**
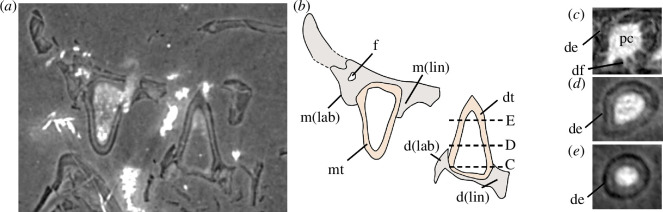
Transverse section of left dentary and maxilla of µCT scan of BP/1/720 Individual II, holotype of *Milleropsis pricei*. (*a*) Transverse section; (*b*) diagrammatic representation of the scan slice; (*c–e*) coronal slices of maxillary teeth moving apically demonstrating plicidentine infolding. Abbreviations: de, dentine; d(lab), labial alveolar shelf of dentary; d(lin), lingual alveolar shelf of dentary; df, dentine folding; dt, dentary tooth; f, foramen; m(lab), labial alveolar shelf of maxilla; m(lin), lingual alveolar shelf of maxilla; mt, maxilla tooth; pc, pulp cavity.

The premaxilla of *Milleropsis* bears five alveoli, indicating that five premaxillary teeth were present, although only four crowns are preserved (figure 4*d*). The premaxillary teeth are conical, isodont and are weakly recurved distally. The teeth have plicidentine infolding forming grooves near their bases (similar to the maxillary dentition, figure 8*c–e*). This ‘base-only’ plicidentine morphology is similar to the condition described in the stem-amniote *Seymouria* [65] as well as early diverging amniotes, including: ophiacodontids [66], sphenacodontids [67], captorhinids [68] and the neodiapsid *Youngina capensis* [69]. This differs from the widespread occurrence of ‘whole-crown’ plicidentine in many non-amniote members of the tetrapod total-group [70–72], some acleistorhinid stem reptiles (e.g. *Colobomycter* [73], *Carbonodaraco* [74]) and ichthyosaurs [75]. Dental implantation in *Milleropsis* is subthecodont, with the labial and lingual walls of the premaxilla being similar in height (figure 8*a–c*), unlike some lepidosauromorphs in which the labial wall extends substantially farther apically, a characteristic of pleurodont dentition [76–78].

#### 4.1.2. Maxilla

The maxilla is the longest element in the snout, extending posteriorly from the midlength of the external naris to the level of the posterior one-third of the orbit (figure 2*b*). Six maxillae are present in the tomography data, many of which are damaged, but the morphology can be accurately reconstructed using the available elements. The right maxilla in Individual IX is the most complete anteroposteriorly, but much of the lateral surface and contribution to the nares is missing. In Individual VI, the dorsal process of the right maxilla (the most posteriorly complete maxilla) is distinctly taller (figure 3*a*), completely overlapping the lacrimal at the level of the 11th alveolus, approaching, but not contacting, the prefrontal. This maxilla was not mechanically exposed and thus not damaged during preparation, unlike the left maxilla of the holotype (figure 2*b*). The main body of the maxilla of *Milleropsis* comprises a dorsal process that forms much of the lateral snout and excludes the lacrimal from the external nares, a subnarial process that attenuates anteriorly and overlies the subnarial ramus of the premaxilla, where it forms the posteroventral margin of the external naris (figure 4*b*), and a suborbital ramus that makes a posterior contact with the jugal.

The dorsal process of the maxilla is dorsoventrally low at the level of the anterior margin of the orbit, and rises anteriorly, attaining its maximum height at the level of the 11th alveolus (figure 3*a*) where it contacts the nasal. The maxillary–nasal contact prevents a contribution of the lacrimal to the naris (visible externally in the holotype individual and in referred specimens) (figures 3*a* and 5*c*), similar to *Eunotosaurus africanus* [39] and *Milleretta rubidgei* [38] (Jenkins *et al*., in review; personal observation BP/1/3822). A lacrimal contribution to the naris is ancestral to Reptilia and present in early diverging stem reptiles such as araeoscelidians [3] and *Orovenator* [15]. In contrast, the lacrimal is excluded from the external naris in procolophonoids (e.g. *Saurodektes kitchingorum*; [55]), neodiapsids [57] and other millerettids. The lateral surface of the dorsal process of the maxilla is weakly concave anteroposteriorly just anterior to the orbit, such that the alveolar surface bows out laterally as it approaches the external naris and also in the suborbital region (figure 3*d*). The lateral surface of the maxilla preserves a row of supralabial foramina along its entire length, with at least eight foramina present (figure 4*a*). The anteriormost foramen (the anterolateral maxillary foramen *sensu* [5]) is the largest, a feature widespread in many Permian and Triassic reptiles (e.g. *Prolacerta*, [79]).

**Figure 9.**
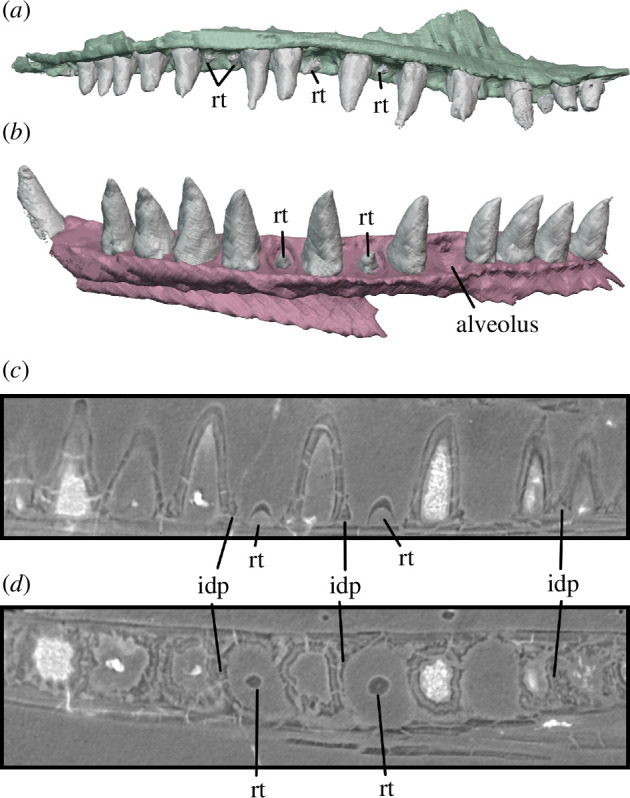
Maxilla and dentary of BP/1/720, holotype of *Milleropsis pricei*. (*a*) Medial view of left maxilla of Individual II showing alternating tooth replacement; (*b*) medial view of right dentary of Individual IX showing alternating tooth replacement, where present; and (*c*) sagittal and (*d*) transverse sections through right dentary showing tooth replacement and implantation. Abbreviations: idp, interdental plate; and rt, replacement tooth.

**Figure 10.**
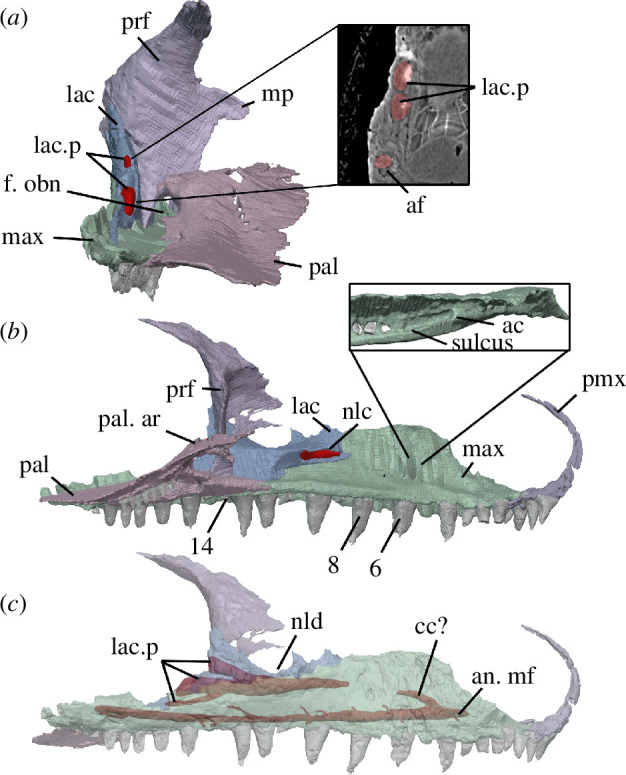
BP/1/720, holotype of *Milleropsis pricei*. Segmented elements from µCT scan of Individual II and premaxilla added from Individual IX focusing on the antorbital region, nasolacrimal duct and maxillary canals. (*a*) Posterior view of segmentation of anterior orbit with pop-out demonstrating the lacrimal puncti and alveolar foramen; (*b*) medial view of segmentation of antorbital region; and (*c*) opaque medial view of segmentation of antorbital region. Abbreviations: ac, alveolar canal; af, alveolar foramen; an. mf, anterior maxillary foramen; cc, possible conical cavity; f. obn, foramen orbitonasale; lac, lacrimal; lac. p, lacrimal puncti; max, maxilla; mp, medial process of prefrontal; nlc, nasolacrimal canal; nld, nasolacrimal duct; pal, palatine; pal. ar., ascending ramus of palatine; and prf, prefrontal.

The maxilla of *Milleropsis* bears 21 alveoli, three more than described by Gow [25]. The maxillary teeth are identical in shape to those of the premaxilla, being conical, slightly recurved, lacking carinae and bearing plicidentine grooves or infolding near the base of the crown (figure 8). Active tooth replacement is evident in five of the 21 preserved alveoli of the left maxilla of Individual II, with the apices of five teeth emerging from their respective alveoli, directly rootward to the primary tooth (figure 9*a*). These replacement teeth originate basal to the erupted tooth, close to the centre of the alveolus. Tooth replacement (when occurring) appears to be alternating, with active replacement and empty sockets occurring at every other tooth position, with some discrepancies posteriorly (figure 4). The alveolar tooth row sits in a series of shallow sockets rootward to the alveolar shelf. The labial wall of the alveolar tooth row extends slightly farther ventrally than the lingual wall (figure 10*a,b*), although not to the extent seen in taxa that possess sub-pleurodont or pleurodont dentitions, such as the lepidosauromorphs *Paliguana* and *Marmoretta* [77,78]. Thin laminae of alveolar bone separate shallow alveoli or sockets, and therefore the dental implantation of *Milleropsis* can be categorized as ‘subthecodont’ [80–82]. The dentition is homodont, as in most neoreptiles [19,83] lacking a caniniform tooth or region. This differs from most synapsids [59], protorothyridids [84], owenettids [55], acleistorhinids [85] and specimens referred to the millerettid *Broomia perplexa* [86], which possess enlarged ‘caniniform’ teeth or a caniniform region. The maxillary teeth of *Milleropsis* decrease in size posteriorly, and the last three teeth are the smallest in both apicobasal height and circumference and are slightly smaller than the premaxillary teeth.

The maxillary alveolar shelf extends medially from the body of the maxilla. The alveolar shelf lacks a medial swelling or supracanine buttress, unlike the condition in some early synapsids [59], *Orovenator* [15] and the archosauromorph *Prolacerta* [56,87,88]. The maxillary alveolar shelf of *Milleropsis* maintains a consistent dorsoventral height along most of its length, up to the level of the third alveolus, from where it thins anteriorly and forms the premaxillary process of the maxilla (figure 9*a*). The anterior foramen for the dorsal alveolar canal is positioned dorsal to the sixth alveolus on the alveolar shelf (figure 10*b*). A well-defined sulcus extends posteriorly from this foramen, extending to the level of the 14th alveolus, when the medial wall of the sulcus begins to merge with the alveolar shelf. The foramen orbitonasale, i.e. the opening for the ophthalmic nerve (CN V_I_), opens at the junction of the maxilla, palatine and prefrontal (figure 10*a*) similar to other stem reptiles such as *Youngina* (personal observation BP/1/2871).

The suborbital ramus of the maxilla of *Milleropsis* takes the form of a low, tapering ramus that terminates anterior to the posterior margin of the orbit at two-thirds of the orbit length (figure 3*a*), as also present in *Orovenator* [15], many non-caseasaurian synapsids (e.g. *Ophiacodon* or *Edaphosaurus* [59]), mesosaurs [89], procolophonians (e.g. *Nyctiphruretus* [90]) and neodiapsids [57]. This differs from the condition in early tetrapods (e.g. [91,92]) and some early diverging amniotes including basal captorhinids (e.g. *Thuringothyris* [93]), acleistorhinid stem reptiles (e.g. *Acleistorhinus* [11]), caseasaurian synapsids (e.g. *Eothyris* [49]) and varanodontines [59] in which the suborbital ramus of the maxilla terminates posterior to the posterior margin of the orbit. This low suborbital ramus obscures the medial contact of the lacrimal and jugal in lateral view and therefore contributes to the orbital margin. The suborbital ramus of the maxilla attenuates posteriorly, approximately at the level of the anterior margin of the orbit (figure 3*a*). At the level of the 17th alveolus, the lateral surface of the suborbital ramus becomes dorsolaterally inclined at an angle of 10–15° from vertical, similar to what has been described in the possible early varanopids *Orovenator* and *Archaeovenator* [15,94,95].

#### 4.1.3. Septomaxilla

The septomaxillae of many early tetrapods are poorly known, in part due to the small size of this bone and its position within the external naris, where it often remains embedded in matrix. A partial left septomaxilla is preserved in the external naris of Individual II and is represented by a fragmentary sheet identified here as the dorsal process (figure 2*a,b*). Fortunately, both septomaxillae are present in Individual IX and remain in articulation within the external nares (figure 4). The complete septomaxilla of *Milleretta* consists of a sheet-like ventral plate (which articulates with its antimere anteriorly), an anterior process that rests on the palatal process of the premaxilla, and a broad, dorsal process that contacts the supranarial process of the premaxilla and the contralateral septomaxilla, partitioning the external nares (figure 7*c,d*).

The septomaxilla of *Milleropsis* lies medial to the snout, lacking any superficial exposure dorsally or laterally, similar to the morphology present in *Orovenator* [15] and acleistorhinids [96]. This contrasts with the more conical septomaxilla of stem amniotes such as captorhinids and recumbirostrans, in which the septomaxilla often forms the entire posterior margin of the external naris [97]. In various synapsids, such as the sphenacodontian *Dimetrodon* or the therapsid *Ictidostoma*, the septomaxilla contributes both to the posterior margin of the external nares and the lateral surface of the snout [98]. The septomaxillary canal of *Milleropsis* is located near the junction of the ventral plate and the dorsal process and is formed by two, anteroposteriorly oriented laminae (figure 7). The dorsal process of the septomaxilla is inclined dorsomedially, roofing the septomaxillary canal anteriorly and contacting the ventral surface of the supranarial process of the premaxilla at about the level of the 3rd premaxillary tooth, at approximately the midlength of the external naris. The dorsal process of the septomaxilla in *Milleropsis* bears an anterior projection similar to the intranarial process present in many non-mammalian synapsids [98].

#### 4.1.4. Nasals

The nasals of all individuals within BP/1/720 are badly preserved and were damaged during initial preparation such that most of the external surface could not be visualized in the scanned specimens. Thin portions of the nasals are present in Individual II, revealing their contact with the dorsal process of the maxilla, and a short portion of the nasal contribution to the external naris. Other information on the nasal of *Milleropsis* is available from the acid-prepared specimen, Individual I [20], and referred specimens (figure 5*b–d*) that were used in our reconstruction (figure 6).

The nasal of *Milleropsis* is an anteroposteriorly short bone, contacting the supranarial process of the premaxilla anteriorly, the maxilla and lacrimal anteroventrally, the prefrontal laterally and the frontal posteriorly. The nasal contributes to the external naris at approximately the level of the fifth maxillary alveolus where it contacts the dorsal process of the maxilla, thereby excluding the lacrimal from the external naris (figure 5*a*). The nasal of *Milleropsis* bears no lateral expansion, and there is no evidence of a narial shelf. The dermal sculpting of the nasal in Individual 1 is poorly developed, lacking the raised bosses or cranial osteoderms that are present on the parietals and frontals, differing from the millerettid *Milleretta rubidgei* in which dermal sculpturing extends anteriorly onto the nasal [25].

#### 4.1.5. Lacrimal

Individual II preserves both lacrimals, although the right lacrimal has been almost entirely damaged by preparation. The following description is based on the left lacrimals of Individuals II and VI, which are missing their anteriormost margins and some of the lateral surface due to preparation damage, as well as the complete left lacrimal of Individual I and SAM-PK-K10082 (figure 5*a,c*). The lacrimal of *Milleropsis* contacts the prefrontal and nasal dorsally, the maxilla ventrally and the jugal posteromedially. The lacrimal of *Milleropsis* is an anteroposteriorly short element, with an anterior process forming slightly less than half of the lateral surface of the snout, and a posteroventral process forming the anteroventral margin of the orbit medially.

The anterior process of the lacrimal comprises a mediolaterally thin lamina that is thickened ventromedially where it houses multiple pathways of the nasolacrimal duct (i.e. nasolacrimal ducts *sensu* [99], figure 10*a*). The lateral surface of the anterior process of the lacrimal slopes dorsolaterally at an angle of about 10° relative to the maxillary tooth row, thereby accommodating the lateral expansion of the prefrontal relative to the tooth row (figure 10*a*). The ventral portion of the lacrimal is overlapped laterally by the maxilla for most of its length, forming a scarf joint. The morphology of this contact is poorly documented in most early amniotes, but it contrasts with taxa such as *Orovenator*, in which there is little overlap between the lacrimal and maxilla [15]. The anterior process of the lacrimal ends abruptly at the level of the seventh or eighth maxillary alveolus where the dorsal process of the maxilla contacts the nasal. The lacrimal is therefore excluded from the narial margin, as reconstructed by Carroll [100] but differing from the reconstructions of Watson [20] and Gow [25], although both studies expressed uncertainty about the anterior extent of the lacrimal. The exclusion of the lacrimal from the external narial margin in *Milleropsis* is similar to mesenosaurine varanopids (e.g. *Mesenosaurus* [40]; *Heleosaurus* [73]), procolophonoids [101] and the ‘neodiapsid’ *Lanthanolania* [102] in which the lacrimal does not contribute to the external naris. This differs from acleistorhinids, in which the lacrimal contribution to the external naris is hidden in lateral view by a dorsal process of the maxilla [103], but the lacrimal still contributes to the external naris internally. This also differs from the plesiomorphic condition in most early amniotes in which the lacrimal contributes to the external naris and is visible in lateral view, as present in stem amniotes (*sensu* Jenkins *et al*., [38] in review) such as protorothyridids (e.g. *Protorothyris archeri* [84]); and captorhinids (e.g. *Labidosaurikos* [104]), early synapsids including varanodontines (e.g. *Aerosaurus* [44]) and caseasaurs [49], and bolosaurid ‘parareptiles’ (e.g. *Bolosaurus* [105]) and pareiasaurs [106].

The posteroventral process of the lacrimal forms the anteroventral margin of the orbit, curving posteroventrally in lateral view. The posteroventral process extends posteriorly along the ventral margin of the orbit and contacts the anterior process of the jugal (figure 2*c*). This contact is obscured in lateral view by a maxillary contribution to the orbital margin (figure 2*b*), similar to other stem reptiles, including acleistorhinids [96], the owenettid *Saurodektes* [107], other millerettids [25] and the neodiapsids *Coelurosauravus elivensis* [108] and *Claudiosaurus germaini* [57]. In contrast, a lacrimal–jugal contact that is visible in lateral view and that excludes the maxilla from the anteroventral margin of the orbit is present in many other early reptiles (or stem-amniotes) including the protorothyridid *Protorothyris archeri* [84], the captorhinid *Moradisaurus grandis* [109], the procolophonid *Procolophon trigoniceps* [64] and most non-saurian neodiapsids [56].

Two large openings for the nasolacrimal duct open posteriorly within the conjunctival groove of the margin of the orbit, best visible in slices of Individual II (figure 10*a*). A third, smaller foramen opens on a medial groove near the posteriormost extent of the lacrimal, not visible in lateral view; this foramen marks a canal that joins the nasolacrimal duct anteriorly, similar to what has been described in *Orovenator* [15] and *Captorhinus laticeps* [43]. The presence of three nasolacrimal foramina may also have been overlooked in many other taxa for which tomography data is not available and the presence of only two foramina is often assumed (e.g. [52,56,110]). The three internal canals converge anteriorly at the level of a pronounced medial expansion of the lacrimal (figure 10*c*). This medial expansion of the lacrimal in *Milleropsis* roofs the anterior pathway of the nasolacrimal duct and it is likely that the duct continued forward as a groove on the medial surface of the maxilla, as in *Milleretta* [25].

The lacrimal of *Milleropsis* does not contribute to the medial flange of the prefrontal (‘antorbital buttress’ *sensu* [111]), similar to the condition in owenettids, among taxa with a broader, medial flange of the prefrontal [55]. In contrast, the bolosaurid *Belebey* [61] and later branching procolophonids [112] possess a medial, triangular flange that contributes to the antorbital buttress. Additionally, there is no medial contribution of the lacrimal to the foramen orbitonasale in *Milleropsis* (figure 10*a*).

#### 4.1.6. Prefrontal

Three nearly complete prefrontals are evident in the scans of BP/1/720, including the left prefrontal of Individual II and both prefrontals of Individual VI. The anterior extent of the dorsal exposure of the prefrontal is unknown in Individual II due to damage to the skull roof. However, the anterior process of the prefrontal of Individual I and referred specimens continues anteriorly to nearly the level of the dorsal process of the maxilla (figure 5). The prefrontal of *Milleropsis* is a triradiate bone, consisting of an anterior process forming the posterior portion of the snout, a dorsal process forming much of the anterodorsal margin of the orbit and a ventromedial process forming much of the anterior orbital rim. The prefrontal contacts the nasal anteriorly, the palatine and lacrimal ventrally and the frontal posterodorsally. The lateral surface of the anterior process lacks both cranial osteoderms and dermal sculpting.

The ventral process of the prefrontal forms the anterior margin of the orbit and is mediolaterally expanded. This process makes contact with the ascending process of the palatine ventrally (figure 10*a,b*), although the contact between these two elements is narrow. Prefrontal contact with the palatine has often been considered to be a synapomorphy of Parareptilia [5], but recent work has demonstrated the presence of this contact in many other taxa, including the dissorophoid temnospondyl *Cacops* [113], the seymouriamorph *Seymouria* [114] and recumbirostrans (*Quasicaecilia texana* [47]), and it is widespread among early amniotes including the synapsids *Edaphosaurus* [59,115] and *Mesenosaurus* [40], the neoreptile *Orovenator* [15], the neodiapsid *Youngina* (personal observation BP/1/2871) and saurians (e.g. the rhynchocephalian *Clevosaurus brasiliensis* [116]) and the early rhynchosaur *Mesosuchus brownii* [117]. Nevertheless, the flange-like morphology of the ventral process of the prefrontal of *Milleropsis* is potentially diagnostic of some ‘parareptile’ subgroups. A mediolaterally expanded prefrontal is present in procolophonids (most prominently in leptopleuronines [118]), and the bolosaurid *Belebey* [61] although the ventral process is narrow in the bolosaurid *Eudibamus* (figure 3*a*; [119]), acleistorhinids (e.g. *Delorhynchus* [120]) and the mesosaur *Mesosaurus tenuidens* [89,103]. The ventral process of the prefrontal of *Milleropsis* forms the lateral margin of the foramen orbitonasale (figure 10*a*). A tongue-like medial process of the prefrontal is present in Individual II of BP/1/720 (figure 10*a*), a feature otherwise reported in procolophonoids [55]. This medial process is absent in other known millerettids including *Milleretta rubidgei* (personal observation BP/1/3822) and is probably autapomorphic for *Milleropsis*.

**Figure 11.**
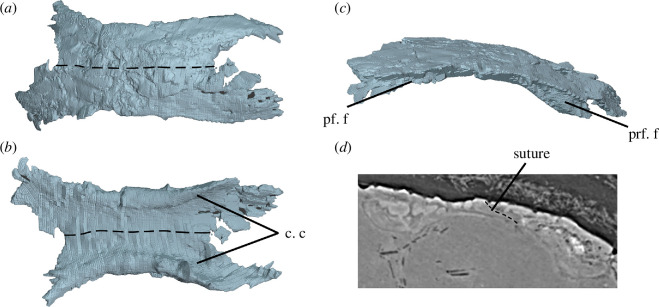
BP/1/720, holotype of *Milleropsis pricei*. Frontals of Individual VI in: (*a*) dorsal; (*b*) ventral, (*c*) right lateral views; and (*d*) transverse section demonstrating sutural relationships of the paired frontals and the lack of ventral flanges for an unossified orbital cartilage. Dashed lines indicate approximate location of the suture between both frontals. Abbreviations: c. c, crista cranii; pf. f, facet for postfrontal; and prf. f, facet for prefrontal.

The posterodorsal process of the prefrontal is a thin, tapering process that forms the anterodorsal margin of the orbit (figure 3). A similar, spur-like posterodorsal process of the prefrontal is widespread among early amniotes, notably captorhinids (e.g. *Euconcordia cunninghami* [52]), mesenosaurine varanopids [40], owenettids [55], early procolophonids [121] and archosauromorphs (e.g. *Prolacerta broomi* [79]). In contrast, a spur-like posterodorsal process is absent in some non-amniotes (e.g. *Seymouria baylorensis* [63]; *Limnoscelis* [48]), possibly due to the presence of a prefrontal–postfrontal contact, and a greater contribution to the skull roof of these elements. The posterodorsal process of the prefrontal of *Milleropsis* fits into a laterally facing facet on the frontal and does not contact the postfrontal posteriorly (figure 11*c*). The prefrontal extends laterally, slightly overhanging the lateral surface of the skull, similar to mesenosaurine varanopids [40]. The lateral expansion of the prefrontal in *Milleropsis* as well as in the millerettid *Milleretta rubidgei* differs from mesenosaurines, however, in forming a more obtuse angle [25].

### 4.2. Skull roof and orbital region

The skull roof of *Milleropsis pricei* is badly preserved, with many elements preserved solely as skims of bone lacking evidence of their original contacts with other elements particularly in holotype Individual II. The descriptions below are mostly based on the more complete (although still damaged) skull roof of Individual VI and the acid-prepped, isolated cranial elements present in Individual I. The skull roof is mildly convex, forming a dorsoventral apex at approximately the midlength of the frontals. The skull roof slopes gently posteroventrally posterior to the orbit, and more strongly anteroventrally anterior to the orbit. The postorbital surface is transversely flat and does not slope ventrolaterally. The parietal is not embayed laterally for a supratemporal fenestra, although there is a ventrolateral flange of the parietal in this region, which led to the hypothesis that millerettids may have ancestrally possessed an upper temporal fenestra [20].

#### 4.2.1. Cranial ornamentation

**Figure 12.**
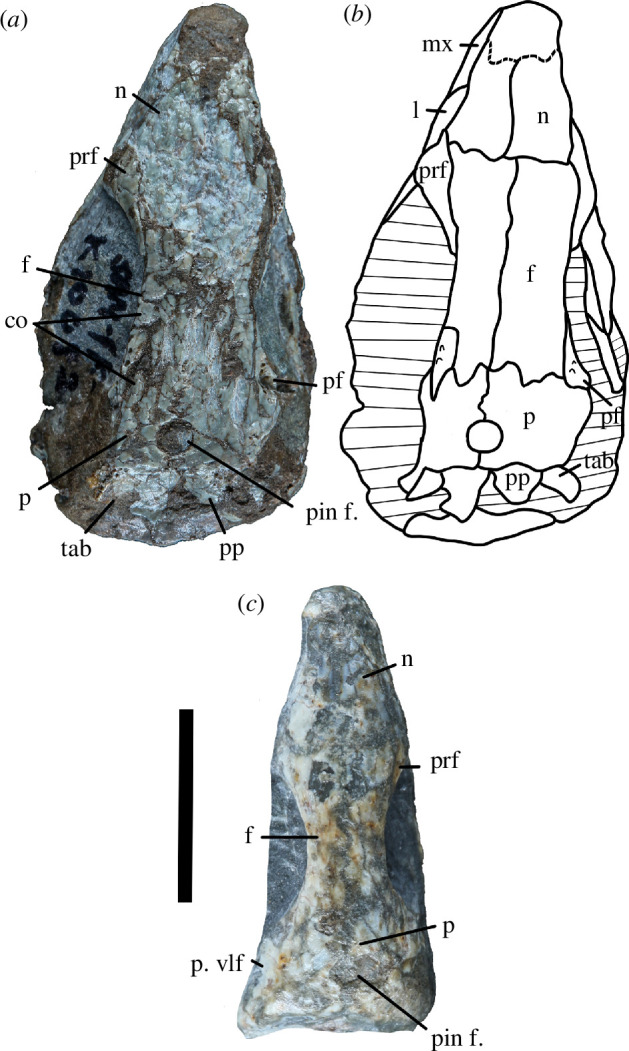
Dorsal view of referred specimens of *Milleropsis pricei*. (*a*) SAM-PK-K8609 and (*b*) line reconstruction; and (*c*) SAM-PK-K10082. Cross hatching indicates damage or matrix. Abbreviations: co, cranial osteoderms; f, frontal; l, lacrimal; m, maxilla; n, nasal; p, parietal; pin. f, pineal foramen; pf, postfrontal; pp, postparietal; prf, prefrontal; p. vlf, ventrolateral flange of parietal, and tab, tabular.

The presence of cranial osteoderms could not be unequivocally determined in our tomography data. However, a poorly preserved dermal skull roof of an individual that corresponds with Watson’s description of Individual VII (although the original labelling is missing) possesses dermal sculpting consisting of low bosses and knobs interspersed by shallow grooves, and identical dermal sculpting is present in referred specimens (e.g. SAM-PK-K8609). This dermal sculpting is restricted to the frontals and parietals, unlike in *Milleretta* and *Eunotosaurus* (personal observation CM-777) in which distinct cranial osteoderms extend onto the postorbitals, jugal and squamosal [25]. A similar condition, in which dermal osteoderms (or a similar ossification of sculpted bone) rest upon the skull roof (but not the lateral skull), is present in referred specimens SAM-PK-K10082 (figure 12) and SAM-PK-K8609 (figure 12*b*) as well as the early diverging millerettid *Broomia* [86].

#### 4.2.2. Frontal

Three frontals are preserved in the scanned block of BP/1/720, the left frontal of Individual II and both frontals of Individual VI, which were segmented as a single element due to a lack of contrast in this region (figure 11*d*). Two additional frontals are present in Individual I. The frontals are anteroposteriorly elongated (they are the longest elements in the skull roof), paired elements and are observed clearly in Individual VI, although the contrast of data in this region was poor (figure 11*d*). The frontals contact the nasals anteriorly and the prefrontals anterolaterally, extending onto the antorbital region of the snout. They contact the parietals posteriorly and the postfrontals posterolaterally (figure 3*c*).

In Individual I, the suture between the frontal and the nasal is oriented approximately parasagittally. In this specimen and SAM-PK-K8609, a thin process of the frontal appears to be overlapped by the nasal, although this cannot be confirmed in any of the scanned individuals. The lateral surface of the frontal bears a distinct, ventrally oriented groove for articulation with the prefrontal, forming a tongue-and-groove joint (figure 11*c*). The frontals have a broad contribution to the dorsal margin of the orbit and lack the ‘lateral lappet’, a distinct lateral extension between the prefrontal and postfrontal that is present in some early amniotes. The absence of a lateral lappet in *Milleropsis* is shared with araeoscelidians [3], mesosaurs (e.g. *Stereosternum* [122]), bolosaurids [49] and most neodiapsids (e.g. *Acerosodontosaurus* [123]). This is in contrast to many other early amniotes, such as acleistorhinid stem reptiles [124], early synapsids such as caseasaurs [49], ophiacodontids [59], sphenacodontids (e.g. *Sphenacodon* [125]) and therapsids (e.g. *Biseridens qilianicus* [126]), some captorhinids (e.g. *Labidosaurus* [127]) and the neodiapsid *Claudiosaurus germaini* [57], in which the lappet is present. In Individual VI, the left frontal overlies the right frontal, forming a scarf joint (figure 11*d*). The short posterolateral processes of the frontals extend posteriorly onto an emargination of the parietal, similar to the interpretation by Watson [20]. This is not shown in the three-dimensional model (figure 3) due to a lack of contrast in this region. The medial contact between the frontal and parietal is a simple and transverse overlapping suture.

The ventral surface of the frontal bears well-developed crista cranii (subolfactory processes of [128]), which extend anteriorly onto the ventral surface of the prefrontal and posteriorly onto the postfrontal (figure 11*b*). Well-developed crista cranii are present in all early amniote lineages [19,59].

#### 4.2.3. Parietal

The paired parietals of Individual II are badly prepared such that only isolated skims and impressions of the bone are observable, and thus were not digitally reconstructed (figure 1). Individual VI possesses both parietals, although much of the dorsal surface has weathered away and the contrast of the data in this region was poor (figure 3). As such, the parietals of Individual VI as reconstructed in the three-dimensional model are missing much of their occipital surfaces, including the posteroventral processes, giving it a falsely ‘square’ posterior margin in figure 3*c*. Watson [20] described the sutural relationships of parietals based on the exposed morphology of Individual VII. Unfortunately, this individual and its counterpart could not be relocated during the present study. Our reconstruction (figure 6) is based on the segmented parietals of Individual VI and referred specimens SAM-PK-K8609 and SAM-PK-K10082 (figure 12). The parietals of *Milleropsis* bear a prominent anterolateral process that separates the postfrontal from the postorbital in dorsal view, an anterior process that contacts the frontals, and a short, posteroventrally inclined occipital portion that contacts the supratemporals, tabulars and postparietals.

The anterolateral parts of the parietals of *Milleropsis* are overlapped by the posterolateral processes of the frontals (figure 3*c*), although the exact sutures between these elements cannot be unequivocally determined. The parietals and frontals appear to form an approximately straight suture, lacking the long, anteromedial process of the parietal visible in *Orovenator* [15] or the neodiapsids *Youngina* and *Claudiosaurus* (personal observation BP/1/3859 [57]). The parietals of *Milleropsis* extend anterolaterally at the level of the frontal–parietal contact, partially separating the postorbital and postfrontals in dorsal view (figure 3*c*). These anterolateral processes of the parietals were described as a feature of Millerettidae by Romer [19] and Watson [20]. However, similar anterolateral processes are also present in some other amniotes, such as the ophiacodontid *Varanosaurus* [129], mesosaurids (e.g. *Mesosaurus* [89]) and captorhinids (e.g. holotype of *Euconcordia cunninghami,* KUVP 8702a [52]), indicating that they are more widespread. In contrast, the parietals of most other early amniotes lack an anterolateral process, and the suture between the postfrontal and postorbital is uninterrupted (e.g. *Prolacerta broomi* [79], figure 6).

The lateral surfaces of the parietals of *Milleropsis* are bounded by contact between the postorbital and squamosal (figure 3*a*). There is no lateral embayment or free lateral margin of the parietal. Therefore, *Milleropsis* lacks an upper temporal fenestra, differing from *Orovenator* [15] and neodiapsids, including crown reptiles [130–132]. This also differs from araeoscelidians [3], which possess an upper temporal fenestra of uncertain homology, and from edaphosaurids in which the parietal has a free lateral margin due to the development of an enlarged lateral temporal fenestra [59]. However, as noted by Romer [19] and Watson [20], millerosaurs (which, at the time included the Millerettidae and *Mesenosaurus*), possess a unique ventrolateral flange that rests on a transverse facet of the squamosal and postorbital as opposed to suturing with these elements (figure 12*b*). Watson [20] interpreted this ‘free lateral margin of the parietal’ as evidence that the millerettids ancestrally possessed an upper temporal fenestra that had become secondarily closed. Interestingly, a similar deflection (or ‘ventrolateral flange’) of the lateral surface of the parietals has also been described in *Eunotosaurus* [133] and the varanopid *Mesenosaurus* [20]. The ventrolateral flange of the parietal of millerettids overlies an unossified gap, which may have influenced later authors’ interpretations ([46] for *Eunotosaurus*; [134] for *Mesenosaurus*) that these taxa were anatomically diapsid due to damage to the parietal in this region.

The dorsal surface of the parietal is plate-like and is horizontal across its full lateral breadth (figure 3). This is similar to the condition in most other early amniote or non-amniote lineages, where the dorsal surface of the parietals is approximately flat. This contrasts with later branching neodiapsids in which the lateral margins of the parietals are deflected ventrolaterally (e.g. *Claudiosaurus* [57]), although early diverging neodiapsids such as *Youngina* possess a similarly flat parietal [8].

A pineal foramen is present on the midline suture between the parietals in *Milleropsis,* located near the posterior half of the element. The pineal foramen is subcircular and large, making up approximately 25% of the anteroposterior length of the parietals (figure 3*c*). The pineal foramen sits in an extremely shallow fossa, which is not as developed as the midline depression or concavity in early diverging procolophonoids such as *Nyctiphruretus acudens* [90] or *Colletta seca* [121].

The occipital shelf of the parietals of *Milleropsis* is sheet-like and downturned posteroventrally at an angle of approximately 70°, as evident in Individual VI (figure 3). The occipital shelf bears facets for the postparietals, which would have overlapped the parietals posterodorsally forming a scarf joint, as shown in the reconstruction by Gow [25] based on the isolated, acid-prepared postparietals in Individual I and the referred specimen SAM-PK-K8609 (figure 12). A distinct facet for the tabular could not be distinguished in the tomography data of Individual VI. However, a tabular was present, and is preserved in SAM-PK-K8609 (figure 12).

#### 4.2.4. Postfrontal

Two postfrontals are preserved, a left postfrontal in Individual II and a right postfrontal in Individual VI. The postfrontals are crescentic, bearing an anterior process that contacts the frontal dorsally, a small posterior process that is overlain by the parietal dorsally, and a ventral process that contacts the postorbital posteroventrally (figures 2*b* and 6).

The anterior process of the postfrontal forms the posterodorsal margin of the orbit (figure 2*b*). It attenuates anteriorly, becoming pointed, and thus does not contribute broadly to the skull roof. The postfrontal of *Milleropsis* is similar to those of araeoscelidians (e.g. *Petrolacosaurus* [135])*, Orovenator* [15] and neodiapsids (e.g. *Macrocnemus bassani* [136]), which possess similar, approximately falciform postfrontals with little contribution to the skull roof. This differs from many early or stem amniotes, including diadectids (e.g. *Diadectes* [137]), captorhinids (e.g. *Captorhinus laticeps* [43]) and synapsids (e.g. *Edaphosaurus* [59]), in which the medial extension of the postfrontal contributes broadly to the skull roof [138–140]. The postfrontal does not contact the prefrontal anteriorly, unlike the condition in many non-amniotes, including temnospondyls (e.g. *Dendrerpeton acadianum* [141]) and diadectids (e.g. *Tseajai campi* [142]) in which prefrontal–postfrontal contact prevents frontal contribution to the orbit [143,144]. The medial surface of the anterior process of the postfrontal of *Milleropsis* bears a slight ridge that fits onto a ventrolateral groove on the frontal (figure 11*c*).

The posterior process of the postfrontal is missing in Individual II due to preparation damage. However, it is preserved in Individuals I and VI, which bear a small posterior process very similar to that in *Milleretta rubidgei* [29] in that it is subtriangular (figure 3*c*). This posterior process fits posteriorly on a concave facet on the parietal, thereby excluding postfrontal contact with the supratemporal. This differs from the condition in owenettids [55], in which the postfrontal and supratemporal are in contact. The ventral process of the postfrontal of *Milleropsis* is concave anteriorly, forming the posterodorsal margin of the orbit. It possesses a posteromedially facing facet or groove for articulation with the postorbital and does not contact the jugal ventrally (figure 3*a*).

#### 4.2.5. Postorbital

The left postorbital is preserved in Individual II (figure 2*b*), and the right postorbital is preserved in Individual VI (figure 3*a*). The postorbital is a triradiate element consisting of a thin, anteroventral process that contacts the jugal, a short dorsomedial process that contacts the postfrontal and an anteroposteriorly elongate posterior process that contacts the parietal and squamosal.

The anterior surface of the anteroventral process of the postorbital is concave where it forms a portion of the posterior margin of the orbit. The orbital surface of the anteroventral process forms a transversely oriented sheet due to medial expansion at the posterior margin of the orbit. The anteroventral process bears a facet for the jugal on its posterior surface, best observed in the right postorbital of Individual VI.

The postorbital of *Milleropsis* bears a broadly triangular dorsomedial process that fits in a facet on the postfrontal (figure 3*a*). The presence of a distinct, dorsomedial process on the postorbital is widespread in early amniotes. For example, it is present in the araeoscelidian *Halgaitosaurus gregarius* [145], the synapsids *Varanosaurus acutirostris* [129] and *Secodontosaurus obtusidens* [146] and the neodiapsid *Hovasaurus boulei* [147]. However, a dorsomedial process of the postorbital is notably lacking in stem amniotes including diadectomorphs (e.g. *Limnoscelis* [48]) and seymouriamorphs (e.g. *Seymouria* [148]), as well as captorhinids in which the postorbital lacks any distinct, dorsal expansion and is instead rectilinear in this region (e.g. *Labidosaurus* [149]). The dorsomedial process of the postorbital of *Milleropsis* is overlain by the parietal dorsally, the plesiomorphic condition within Amniota. This differs from the condition in owenettids such as *Saurodektes kitchingorum* [55] and *Candelaria barbouri* [150], in which a posterior extension of the postfrontal prevents parietal–postorbital contact in lateral view. Among neodiapsids, a postorbital–parietal contact has been interpreted as absent in taxa such as *Youngina* [57]. However, our observations of the tomography data of *Youngina* specimen BP/1/2871 suggest that the contact of these elements is obscured in lateral view, but present medially near the anterior border of the upper temporal fenestra.

The posterior process of the postorbital is overlapped by the parietal dorsally and strongly overlaps the squamosal posteriorly, best observed in Individual VI (figures 3*a* and 6). The posterior process of the postorbital is concave ventrally where it forms the dorsal margin of a lateral temporal emargination or arch, as also present in most taxa with temporal fenestrae (i.e. ‘synapsid’, ‘diapsid’ and ‘euryapsid’ conditions [151]), reviewed by [152]. This differs from the situation in procolophonoids in which an anterodorsal process of the quadratojugal and the presence of squamosal–jugal contact prevents the postorbital from contributing to the dorsal margin of the lateral temporal emargination [112]. In the bolosaurids *Bolosaurus striatus* [105] and *Belebey vegrandis* [61], the postorbital is excluded from the dorsal margin of a lateral temporal emargination by squamosal–jugal contact. The posterior process of the postorbital of *Milleropsis* is dorsally convex, confirming evidence from the parietals that there was no upper temporal fenestra. The posterior process narrowly approaches but does not contact the supratemporal posteriorly, similar to *Millerosaurus ornatus* [25], *Orovenator* [15] and neodiapsids [15]. However, this is unlike the condition in *Milleretta* and most ‘parareptiles’ [63] in which supratemporal–postorbital contact is present.

The posterior process of the postorbital of Individual II (the holotype individual) is short and accuminate (figure 2*b*), whereas in Individual VI it is rectilinear and anteroposteriorly elongated (figure 3*a*), as long as most of the posterior temporal region. It is unclear if this is related to individual variation or to differences in ontogeny between the two individuals. It is also likely that the preparation to the temporal region of Individual II has artificially shortened the posterior process.

#### 4.2.6. Postparietal

The paired postparietals are lacking completely in Individual II and are present only as skims of bone in Individual VI. Therefore, the postparietals could not be reconstructed in our three-dimensional model (figures 3 and 6). Individual I preserves both postparietals, which were described as ‘dermosupraoccipitals’ by Watson [20]. Both postparietals are also preserved in SAM-PK-K8609 (figure 12). The postparietals of Individual I were removed during acid preparation by Gow [25] and these isolated postparietals (and those of SAM-PK-K8609) serve as the basis of the description below (figure 12*a*).

The postparietals are subrectangular, paired elements that overlap the occipital shelf of the parietal in dorsal view. Paired postparietals are widespread across early amniotes, being present in early diverging synapsids such as the caseasaur *Eocasea* [153], and among stem reptiles such as the araeoscelidian *Petrolacosaurus* [3] and the neodiapsids *Youngina* [57], *Weigeltisaurus* [154] and tentatively in *Hovasaurus* [147]. This differs from the single, midline postparietal present in eupelycosaurian synapsids such as the varanopid *Varanops* [155], stem reptiles including acleistorhinids such as *Feeserpeton* [103] and *Acleistorhinus* [11], and some procolophonians (e.g. *Nyctiphruretus* [90] but not *Saurodektes* [55]). The postparietal of *Milleropsis* is a relatively small bone of the skull roof; together, the transverse width of both postparietals is approximately equal to 80% of the width of a single parietal [20]. The dorsal surface of the postparietal is shallowly concave. The postparietal contacts its antimere along the midline for its entire length and is not separated by an ascending process of the supraoccipital, unlike early or stem amniotes such as recumbirostran microsaurs [47] and captorhinids [43,156]. This condition in *Milleropsis* is therefore similar to most members of Neoreptilia (*sensu* [15]), including procolophonians (e.g. *Eomurruna* [107]) and neodiapsids such as *Youngina* and *Claudiosaurus* [57].

We accept the description and reconstruction of Gow [25] on the relationships of the postparietal and tabular for BP/1/720 in that the postparietal is probably overlapped by the tabular in what appears to be a butt joint. We also accept Gow’s [25] description that the postparietals of *Milleropsis* overlap the supraoccipital and contribute to the dorsal margin of the post-temporal fenestrae ventrally. The ventral surfaces of the postparietals do not contact the opisthotics, as also seen in early amniotes (e.g. *Araeoscelis* [157]) and stem amniotes (e.g. *Captorhinus* [43]), but contrasting with early tetrapods and some ‘microsaurs’, such as the recumbirostran *Brachydectes* [158].

#### 4.2.7. Tabular

**Figure 13.**
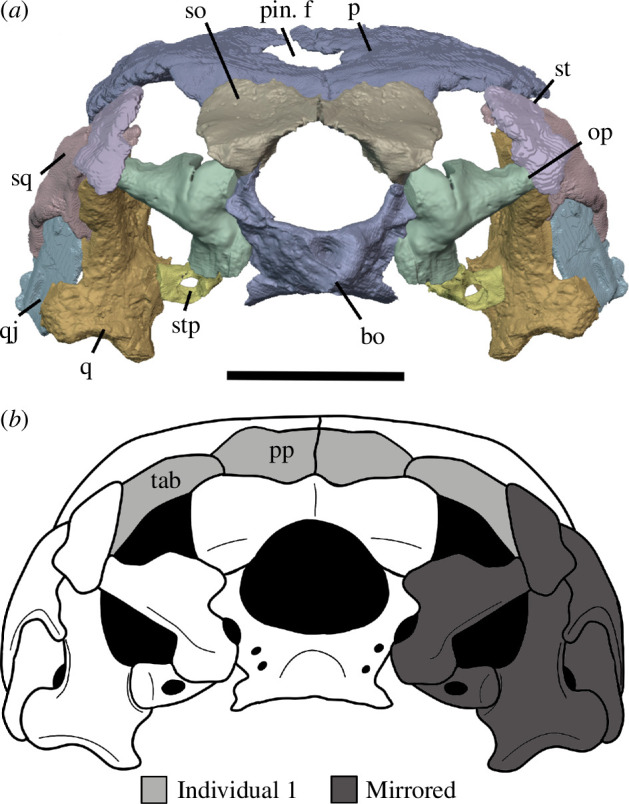
Reconstructions of *Milleropsis pricei* BP/1/720 in occipital view: (*a*) segmentation and (*b*) line drawing. Refer to the key for shading. Abbreviations: bo, basioccipital-exoccipital complex; o, opisthotic; p, parietal; pin. f, pineal foramen; pp, postparietal; q, quadrate; qj, quadratojugal; so, supraoccipital; sq, squamosal; st, supratemporal; stp, stapes; and tab, tabular. Scale bar represents 5 mm.

As with the postparietals, both tabulars are visible in Individual I and SAM-PK-K8609, but are not present in our scans, and thus were not three-dimensionally reconstructed (figure 13). A distinct, ossified tabular is present in many early amniotes but is absent in captorhinids [93], procolophonoids [64] and most neodiapsids (including members of the reptile crown group Sauria) other than the early diverging neodiapsid *Youngina capensis* [25], although the presence of the tabulars in tangasaurids remains unknown [57,147].

The tabular of *Milleropsis* is a slender, narrow bone contributing to the occipital margin of the skull, and is best preserved in SAM-PK-K10082. The tabular sits on a facet on the occipital shelf of the parietal, immediately lateral to the postparietals (figure 12). The posterior end of the tabular contacts and is probably overlapped by the supratemporals, although it is difficult to determine their exact relationship due to damage from acid preparation on Individual I. In *Milleropsis,* contact between the tabular and supratemporal laterally prevents tabular contact with the squamosal, a feature shared with the neodiapsid *Youngina* [56]. This differs from the condition in most other early amniotes [3,59] in which the tabular and squamosal are in contact in lateral or occipital view. The posterior process of the tabular of *Milleropsis* forms the dorsolateral margin of the posttemporal fenestra and attenuates distally at a level dorsal to the supratemporal.

#### 4.2.8. Supratemporal

An incomplete, left supratemporal of Individual II was reconstructed in our three-dimensional model (figure 13). The supratemporal of *Milleropsis* is a narrow bone with little contribution to the skull roof. The maximum transverse width of both supratemporals is about 15% of the skull roof width. Individually each supratemporal is about one-third the width of the postparietals (figure 2*c*). The supratemporal of *Milleropsis* contacts the parietal and squamosal ventrally and the tabular medially, and possibly the paroccipital process of the opisthotic posteromedially.

The supratemporal articulates with the parietal medially, resting in a short trough, similar to the condition in early neodiapsids [56] and eupelycosaurs including varanopids [40]. The supratemporal abuts the squamosal ventrolateral to this trough. However, an unossified gap is present ventral to the supratemporal at the junction of this bone with the parietal and squamosal, which could give the impression of an upper temporal fenestra if the supratemporal were missing due to damage.

The posteroventral extent of the supratemporal in *Milleropsis* exceeds that of the tabular, similar to the varanopid *Heleosaurus* [73] and to stem reptiles including mesosaurs [89] and procolophonians [11], and the early diverging neodiapsid *Youngina* [56]. This is unlike the condition in most early amniotes, including protorothyridids [159], captorhinids [93], araeoscelidians [3] and non-caseid synapsids [153], in which these two bones end at approximately the same level. In *Milleropsis*, the supratemporal overhangs the posterior skull, a feature noted by Watson [20] as diagnostic of Millerosauria.

#### 4.2.9. Jugal

The anatomy of the jugal (and the entire lateral temporal region) of *Milleropsis pricei* has been contentious. It was described as a triradiate bone forming a lower temporal bar by Watson [20], but as a boomerang-shaped bone that does not form a complete lower temporal bar by Gow [25] and subsequent studies. The jugal morphology of the holotype individual, Individual II, which was the focus of both the previous studies, is complicated by preparation damage to the temporal region (figure 1*a*) and it is marked by pockets and holes. Furthermore, this jugal is rotated inwards at an angle of about 90° such that it now lies in the orbit perpendicular to other skull roof elements (figure 2*b*). Our tomography data reveal that both jugals are present in Individual VI (figure 3*a,b*), plus another isolated left jugal identified from the acid-prepped bones of Individual I. The jugal of *Milleropsis pricei* comprises two main processes: an elongate suborbital process that contacts the ectopterygoid, maxilla and lacrimal, and an anteroposteriorly broad dorsal process that contacts the postorbital, forms the anterior margin of the lower temporal emargination and bears a short, posterior spur (figure 6*a*).

The mediolaterally thin suborbital process of the jugal attenuates anteriorly and makes a point contact with the lacrimal just medial to the suborbital ramus of the maxilla. This contact is not visible in lateral view (figure 2*b*). The posterior portion of the suborbital process fits into a shallow recess on the medial surface of the maxilla (figure 2*c*), and it is at this level that the jugal contacts the ectopterygoid (figure 2*d*).

The dorsal process of the jugal is anteroposteriorly broad at its base, although not to the extent of *Milleretta rubidgei*, in which the dorsal process of the jugal extends posteriorly across half of the temporal region in ontogenetically young individuals, and extends further posteriorly, contacting the quadratojugal and squamosal, in adults [25]. The dorsal process of the jugal of *Milleropsis* is overlapped by the ventral process of the postorbital anterodorsally, although there is no distinct facet or groove for this articulation. The dorsal process attenuates dorsally just ventral to the origin of the posterior process of the postorbital (figure 3*a*). The dorsal process of the right jugal of Individual VI (that is unexposed and so was not impacted by preparation) is anteroposteriorly longer than the damaged dorsal process of the left jugal in Individual II, extending farther posteriorly into the lower temporal region.

A small posterior spur, or subtemporal process, is present in the undamaged jugals (Individual VI), in agreement with Watson [20], but this is lacking in the exposed jugals (noted by Gow [25]), probably due to preparation damage. In any case, the subtemporal process is short, and so the jugal probably did not contribute to a complete lower temporal bar, in agreement with Gow [25] but not Watson [20]. This is similar to the condition in *Lanthanolania* and the neodiapsid *Hovasaurus* [102] and to early ichthyosauromorphs (e.g. *Hupehsuchus* [160]; *Cymbospondylus* [161]), which possess a small posterior process of the jugal but lack a fully formed lower temporal bar [147]. It is also similar to early archosauromorphs (e.g. *Prolacerta* [56]), and to the millerettid *Millerosaurus nuffieldi* [25], which possesses a more distinct posterior process yet no lower temporal bar. Among neoreptiles, a lunate jugal that lacks even a small posterior process is present in procolophonians [107, fig. 25] and many non-saurian neodiapsids, such as *Claudiosaurus germaini* [57]. Conversely, the jugal is triradiate, with a prominent posterior process in most other fenestrated amniotes that possess a lower temporal bar (as opposed to emargination), including synapsids (e.g. *Oedaleops campi* [162]; *Ophiacodon* [59]), some araeoscelidians (*Petrolacosaurus* but not *Araeoscelis* [3]), the earliest-known millerettid *Broomia* (although this contact is ambiguous, see [86]), and the neodiapsid *Youngina* (Broom [163]). Although the anterior process of the quadratojugal in *Milleropsis* is damaged, we follow Gow [25] in reconstructing the jugal as not contacting the quadratojugal (figure 6*b*). Furthermore, in the right jugal of Individual VI, there are several bone fragments in the vicinity of the lower temporal emargination that may represent a lower temporal bar (figure 1*a*), but we refrain from assigning these fragments to any element in particular, especially because there are no indications of this morphology in any of the other individuals of *Milleropsis* (e.g. SAM-PK-K 7751, SAM-PK-K8609, figure 5). Nevertheless, all known jugals of *Milleropsis* lack a facet for the quadratojugal, and a complete lower temporal bar was almost certainly absent.

#### 4.2.10. Squamosal

**Figure 14.**
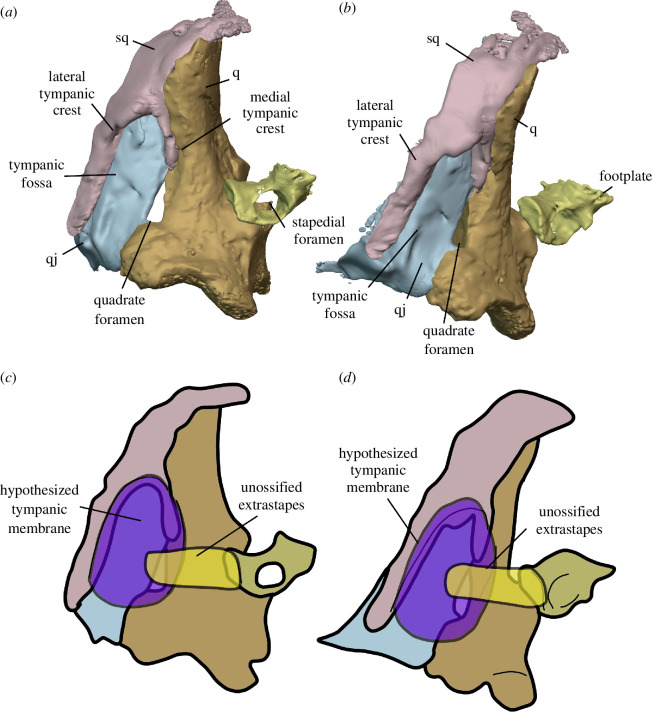
BP/1/720, holotype of *Milleropsis pricei*. Tympanic region of Individual II in: (*a*) posterior, (*b*) posterolateral views and (*c,d*) reconstruction of the tympanic region in posterior and posterolateral views. Abbreviations: qj, quadratojugal; qu, quadrate; sq, squamosal; and stp, stapes.

Both squamosals are preserved in Individual II, the left of which is the more completely preserved, although it is displaced anteriorly along with the quadratojugal. The left squamosal is also present in SAM-PK-K7751 and demonstrates the relationship of this bone with surrounding elements, although it is mediolaterally crushed and missing much of its lateral surface. The squamosal of *Milleropsis* forms a ‘squamosal–quadratojugal complex’ in which these elements strongly overlap and share an emargination or fossa on their posterolateral surfaces (figure 14*b,c*). The squamosal of *Milleropsis* comprises an anteroventral process that frames the lower temporal emargination, an anterodorsal process that contacts the postorbital anteriorly, and a dorsal process that contacts the parietal and supratemporal dorsally (figure 2*b*). The squamosal lacks a strongly developed occipital shelf, exposing the quadrate in posterior view.

An anterodorsal process of the squamosal extends from the ventral portion of the supratemporal posteriorly and to the postorbital anteriorly, based on the reconstruction of Watson ([20], figure 2). This process is not evident in any of our tomography data due to damage in this region, but is visible as several, incomplete skims of bone on the external surface of Individual II that underlie the supratemporal as well as referred specimen SAM-PK-K7751. As preserved, the anterodorsal process appears to end in a short, square-shaped tip, very similar to that reconstructed for *Eunotosaurus africanus* [46]. The anterodorsal process of the squamosal is overlapped by the parietal dorsally, precluding the parietal from bearing a free lateral margin (contra [25], figure 21).

The anteroventral process of the squamosal of *Milleropsis* is anteroposteriorly short, not extending much farther anteriorly than the jaw articulation. The medial surface of this process bears a well-developed, anteroventrally oriented ridge that forms the lateral portion of the articulation with the quadratojugal and contributes to the posterodorsal margin of the lateral temporal opening. More medially, the anteroventral process of the squamosal bears a deep facet, continuing the articulation with the unusually tall dorsal process of the quadratojugal (best seen in anterior or dorsal view, figure 2*c*). The anteroventral process does not contact the posterior margin of the jugal, similar to the condition in *Millerosaurus nuffieldi* [25] and neodiapsids such as *Acerosodontosaurus piveteaui* [164] and *Coelurosauravus elivensis* [108]. This differs from the morphology in other early amniotes such as synapsids (e.g. *Edaphosaurus* [59]), captorhinids (e.g. *Thuringothyris mahlendorffae* [93]), and araeoscelidians (e.g. *Araeoscelis gracilis* [62]), in which squamosa–jugal contact is present. The anteroventral process of Individual II (figure 2) is artificially shortened in our three-dimensional reconstruction due to a lack of contrast in this region, but in the specimen itself (figure 1) it extends ventrally nearly as far as the quadratojugal, ending in a square-shaped tip as shown in our reconstruction line drawing (figure 6*b*).

The dorsal process of the squamosal of *Milleropsis* bears an oval-shaped facet or depression for the overlying supratemporal. The dorsal process then curves posterodorsally, merging into a distinct posterodorsal process that overhangs the dorsal process of the quadrate in lateral view and bears a fossa on its ventral surface for the quadrate (figure 14). A posterodorsal process of the squamosal is additionally present in South African mesenosaurines (Afrothyra *sensu* [165]), other millerettids (e.g. *Milleretta*; Jenkins *et al*., [38] in review), and early neodiapsids [7]. A posterodorsal process is absent in most early amniotes, in which the dorsal process of the squamosal is rectilinear posteriorly, including protorothyridids (e.g. *Protorothyris archeri* [84]), captorhinids (*Labidosaurus hamatus* [149]), araeoscelidians (e.g. *Araeoscelis* [62]), mesosaurids [122], ankyramorphans [11] and synapsids [59]. The presence of a distinct posterodorsal process of the squamosal was considered autapomorphic for Neodiapsida by previous studies (e.g. [7,154]), although its presence in millerettids suggests that this feature may be plesiomorphic to Neodiapsida.

The posteromedial process of the squamosal of *Milleropsis* forms a tympanic fossa or ‘otic notch recess’ of Gow [25], shown here in figure 14, making it distinct from that of other stem reptiles. This tympanic fossa, also present in *Milleretta rubidgei* [25]*,* is formed by the presence of two crests framing an emargination that opens ventrally onto the quadratojugal and quadrate (figure 14*a*). These two crests, hereby ‘tympanic’ crests, consist of a lateral ‘tympanic’ crest that frames the anterior margin of the tympanic fossa and a distinct medial ‘tympanic’ crest that forms the posterior boundary (figure 14*b*). The presence of a tympanic fossa is remarkably similar to the morphology exhibited by procolophonians, including the enigmatic *Nyctiphruretus acudens* [90]*,* the early procolophonoid *Saurodektes kitchingorum* [55] and the nycteroleter *Emeroleter laevis* [166]. Early amniotes have a different condition in which the posterior surface of the squamosal is flat or mildly convex, lacking any emargination or ridge, as exemplified by the sphenacodontid *Dimetrodon milleri* [59], the captorhinid *Captorhinus laticeps* [43], and mesosaurid, bolosaurid and acleistorhinid stem reptiles [11].

The squamosal lacks a broad occipital shelf that overlaps the quadrate posteriorly (figure 14*a*). This absence has also been reported in diadectomorphs [5], varanopids (e.g. *Mycterosaurus* [167]), some araeoscelidians [157], the procolophonian *Nyctiphruretus acudens* ([90], figure 1*e*) and neodiapsids [7]. In contrast, a well-developed occipital shelf is prevalent in most early amniotes such as the caseid synapsid *Euromycter rutena* [168], captorhinids [43] (figure 1), protorothyridids [84] and most procolophonians [64]. The lack of an occipital shelf of the squamosal in *Milleropsis* and other millerettids indicates that this morphology is not restricted to Neodiapsida (contra [7]). The squamosal of *Milleropsis* does not contribute to the occipital region. Therefore, there is also no squamosal contribution to the posttemporal fenestra, another feature millerettids share with neodiapsids among stem reptiles, contrasting with the condition in supposed ‘eureptiles’ such as captorhinids [15].

#### 4.2.11. Quadratojugal

A single, left quadratojugal is preserved in Individual II and a partial right quadratojugal is preserved in SAM-PK-K8609. The quadratojugal of *Milleropsis* consists of a broken anterior process forming the ventral portion of a lateral temporal emargination, an unusually tall dorsomedial process that contacts the squamosal, and a medial process that contributes to the ‘otic notch recess’ of Gow [25] and contacts the quadrate. The lateral surface is extremely abraded due to preparation damage, particularly the anterior process (figure 1).

An anterior process of the quadratojugal was not described by Gow [25], who interpreted there to be a gap separating the quadratojugal from the jugal, therefore resulting in the presence of a ventrally open temporal fenestra (= temporal emargination of [169]). However, a clear anterior process is preserved on Individual II, although much of its anterior-most portion was prepared away after the initial study by Watson ([20]; figure 2*b*). Microscopic observation of the specimen reveals that this process appears to thin anteriorly as it approaches the jugal, a morphology best observed in dorsolateral view. A similar anterior process of the quadratojugal is observed in SAM-PK-K8609. Since both the posterior portion of the jugal and the anterior portion of the quadratojugal are damaged in Individual II, whether these bones were in contact cannot be determined in this specimen. However, we accept the reconstruction of Gow [25], which shows the quadratojugal ending just posterior to the jugal in *Milleropsis* without contacting it, therefore forming a lower temporal emargination, based on the absence of a quadratojugal facet on the jugal and lower temporal bar in all referred specimens (figure 5). The anatomical difference between these two states (ventrally closed lower temporal ‘fenestra’ versus ventrally open temporal emargination) is small and can vary through ontogeny or among closely related species or in some groups including among early neodiapsids, archosauromorphs and squamates [57,79,170].

A quadratojugal foramen passes between the junction of the quadratojugal and quadrate, as in most early amniotes in which this region is observable [43]. The medial process of the quadratojugal bears an emargination on its posterior surface that forms the ventral portion of a probable ‘tympanic’ fossa, as in *Millerosaurus ornatus* [20], *Milleretta rubidgei* [25] and procolophonians [171]. The medial tympanic crest continues ventrally onto the quadrate whereas the lateral ‘tympanic’ crest continues onto the quadratojugal in *Milleropsis*. These crests do not meet ventrally (figure 14*a*). Interestingly, the quadratojugals and quadrate of early rhynchocephalians such as *Gephyrosaurus bridensis* [172] and *Diphydontosaurus avonis* also bear tympanic crests, which are absent in later rhynchocephalians such as *Sphenodon* in which the quadratojugal is reduced or fused with the quadrate [173].

The dorsomedial process of the quadratojugal is well developed in *Milleropsis* and extends most of the dorsoventral height of the temporal region, although this is partially hidden by the squamosal in lateral view. A similarly developed dorsomedial process of the quadratojugal is present in other millerettids including *Milleretta rubidgei* (personal observation BP/1/3822) and *Millerosaurus oratus* [25, fig. 21]. In contrast, the quadratojugal of most early amniotes is dorsoventrally low (e.g. *Captorhinus aguti,* personal observation OMNH 44816)*,* in which the quadratojugal is a short element in the postorbital region. The dorsomedial process of *Milleropsis* fits onto a concave facet on the squamosal posteriorly and forms the posterior margin of a lateral temporal emargination (figure 2*b*).

#### 4.2.12. Quadrate

Both quadrates are well preserved in Individual II (figure 2*a,b*) and an additional left quadrate is present in Individual VI (figure 3*a*). The quadrate consists of a posterodorsally directed shaft, a condylar region and an anteromedially directed lamina forming the pterygoid ramus. The quadrate of *Milleropsis* is overlapped by the quadratojugal and squamosal laterally, and contacts the supratemporal dorsally, paroccipital process dorsomedially and articular ventrally.

The shaft of the quadrate is straight posteriorly and lacks ‘tympanic’ crests present on the quadratojugal and squamosal, although the quadrate probably contributed to the medialmost margin of the tympanic emargination in *Milleropsis,* indicated by a shllow fossa (figure 14*a*). This differs from the condition in many crown reptiles, in which a large emargination or tympanum on the posterior surface of the quadrate is widely present, including in testudines [174] and ‘traditional’ archosauromorphs, and well as lepidosauromorphs which often bear a distinct quadrate conch [130]. The quadrate shaft of *Milleropsis* is inclined posterodorsally towards the occiput, as in procolophonians such as the pareiasaur *Deltavjatia vjatkensis* [175] and the procolophonid *Hypsognathus fenneri* ([176]; figure 14*b*), as well as weigeltisaurid neodiapsids [108]. This differs from the anterodorsal orientation of the quadrate shaft present in the diadectomorph *Tseajaia campi* [142], caseasaurs (e.g. *Eocasea martini* [153]) and ophiacodontids (e.g. *Varanosaurus* [129]). It also differs from other early amniotes in which the quadrate is vertically oriented, including sphenacodontians (e.g. *Sphenacodon ferocior* [177]), captorhinids (e.g. *Captorhinus laticeps* [43]) and neodiapsids (e.g. *Coelurosauravus* [108]). The dorsomedial surface of the quadrate shaft (possibly together with the squamosal or supratemporal) received the distal tip of the paroccipital process of the opisthotic (figure 13). The quadrate of *Milleropsis* lacks the prominent dorsal convexity, head or cephalic condyle of saurians (e.g. the early lepidosauromorph *Paliguana* or the archosauromorph *Macrocnemus* [77,136]).

The pterygoid wing of the quadrate is an anteromedially directed lamina that extends from the condylar region and forms a scarf joint with the quadrate ramus of the pterygoid (figure 2*c*). The ventral margin of the pterygoid ramus of *Milleropsis* is elevated dorsally relative to the condylar region, as in *Edaphosaurus* ([115], figure 12*a*), most early neoreptiles (e.g. *Sauroparieon anoplus* [178], figure 1*b*) and neodiapsids, as noted by Pritchard and Sues [179]. In contrast, most non-neoreptilian taxa, including temnospondyls (e.g. *Acheloma* [180]), recumbirostrans (e.g. *Euryodus* [181]), captorhinids (e.g. *C. aguti*; personal observation OMNH 44816) and araeoscelidians (e.g. *Petrolacosaurus* [3]) have a pterygoid ramus of the quadrate that is level with the condylar region. The condylar region of *Milleropsis* consists of two ventrally facing processes, the quadrate condyles. The medial condyle extends ventral to the lateral condyle, and both articulate with their respective cotyles of the articular bone (figure 14*a*).

A small convex ridge is present on the medial surface of the quadrate, dorsomedial to the condyles and posteroventral to the pterygoid wing. This marks the position of the attachment of the stapes to the quadrate (figure 14*a*). A similar morphology is present in some recumbirostrans [158] and ankyramorph stem reptiles such as *Procolophon* [64], *Feeserpeton* [103] and *Australothyris* (‘medial flange for stapes’ of [73]). Similarly described ‘stapedial bosses’ or ‘stapedial processes’ [182] are present in the early neodiapsids *Youngina* [56], *Claudiosaurus* [57], *Hovasaurus* [147] and *Thadeosaurus* [183], but are absent in weigeltisaurids [184] and saurians, in which the medial surface of the quadrate is flat (e.g. *Macrocnemus bassanii* [136]; *Diphydontosaurus* [173]). This differs from the more rigid stapedial articulation surface present in early amniotes, including synapsids [59,185], captorhinids [186] and araeoscelidians (e.g. *Petrolacosaurus* [3]), in which the distinct medial stapedial depression is surrounded by dorsal and ventral flanges (the ‘stapedial recess’ of [182]).

#### 4.2.13. Scleral ossicles

Scleral elements are not present in the scanned individuals of *Milleropsis*. A large sclerotic ring, nearly filling the orbit, was reported in Watson’s Individual III [20].

### 4.3. Palate and braincase

**Figure 15.**
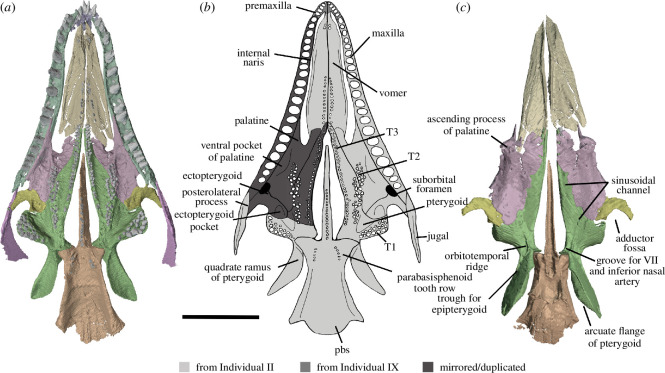
Reconstructions of *Milleropsis pricei* BP/1/720 in palatal and dorsal views. (*a*) Segmentation and (*b*) line drawing in palatal view; (*c*) dorsal view. Refer to the key for shading. Abbreviations: pbs, parabasisphenoid; T1, transverse flange tooth row; T2, anterolateral tooth row of palate, and T3; palatal ramus tooth row. Scale bar represents 5 mm.

All palatal elements are present in Individual II of BP/1/720 and the right elements are complete, although most of the left elements are damaged or missing, except for the left vomer (figure 15). The choanae are anteroposteriorly long and appear very slender, strongly excavating the anterior margin of the palatine. An extremely long interpterygoid vacuity extends anteriorly to the level of approximately the 14th maxillary tooth, separating the pterygoids along their entire length, and extending between the vomers along most of their length. The suborbital foramen is situated between the ectopterygoid posteriorly, the palatine anteriorly and the maxilla laterally. A maxilla contribution to the suborbital opening is present, similar to the condition in other millerettids including *Milleretta* (personal observation BP/1/3822), *Eunotosaurus* [46], and to araeoscelidians and neodiapsid reptiles [3]. In contrast, procolophonians possess a suborbital opening that is restricted to the ectopterygoid and palatine [64]. There is no elongated suborbital fenestra, unlike the condition in some neodiapsids [5].

#### 4.3.1. Vomer

The paired vomers of *Milleropsis* are anteroposteriorly elongated elements, contacting the premaxillae anteriorly, the palatines posteriorly and the pterygoids posteromedially (figure 2*d*). Each vomer bears a single row of teeth on its ventral surface, located medially on a low, longitudinal ridge. Additional, enlarged, paired teeth are present anteriorly, noted as a synapomorphy of millerettids by Gow [25]. The vomers form the medial boundary of the choana and are separated from midline contact for a short distance posteriorly by the palatal process of the pterygoid (figure 15).

Anteriorly, the vomers of *Milleropsis* produce a narrow anteromedian process that contacts the premaxillae. Due to preparation in this region in Individual II, it is difficult to determine the presence or absence of a second, laterally oriented process that would have contributed to an asymmetric vomerine process. However, the vomer of Individual IX appears to lack a clear, asymmetric vomerine process in our tomography data, instead ending as a single, tapering point, where it overlaps the premaxilla. A bifurcated anterior process of the vomer is absent in *Milleretta* and has been proposed as a synapomorphy potentially uniting some varanopids (e.g. *Archaeovenator* [94]) with Neoreptilia (*sensu* [15]).

**Figure 16.**
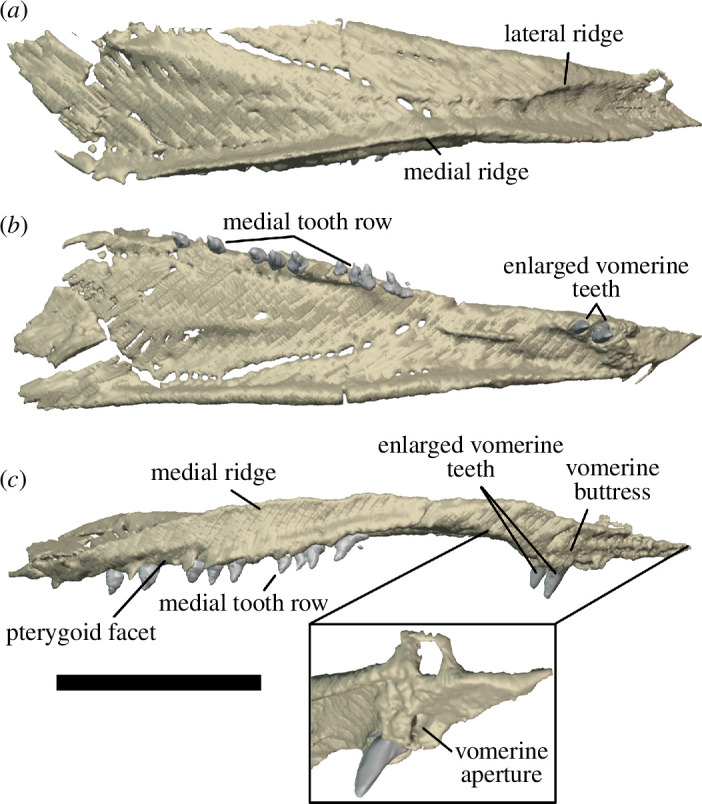
BP/1/720, holotype of *Milleropsis pricei*. Segmented left vomer from µCT scan of Individual II in: (*a*) dorsal, (*b*) ventral view and (*c*) medial view with detail in anteromedial view. Scale bar represents 2 mm.

The lateral margins of the vomer are dorsoventrally thickened, forming the medial border of the choana for its entire length. A median ridge extends anterolaterally along the midline of the dorsal surface of the vomer, as described in *Petrolacosaurus* [3] and *Orovenator* ([15,95]; figure 16*a*). A second, anterolaterally tapering ridge is located laterally to the median ridge. This lateral ridge in *Milleropsis* is weakly developed, as in *Orovenator* [15] and *Youngina* [69]. This differs from the ‘ascending flange’ of the vomer present in early tetrapods such as the recumbirostrans *Euryodus dalyae* ([181], figure 4*b*) and *Llistrofus pricei* ([187], figure 7*a*), as well as captorhinids such as *Captorhinus laticeps* ([43, fig. 24] where it is referred to as the ‘alar projection’) and probably supported the paraseptal cartilage.

The ventral surfaces of the vomers of *Milleropsis* of BP/1/720 are complex, marked by several ridges and concavities (figure 16*b*). A longitudinal ridge on the ventral surface of each vomer adjacent to the midline supports a single, vomerine tooth row, similar to the condition described for *Milleretta rubidgei* [25] and *Mesenosaurus romeri* [40]. This differs from the vomer of many other early amniotes that possess at least two tooth rows on each vomer, including one that extends onto the vomer from the palatine, present in procolophonians such as *Saurodektes* [55] and the neodiapsid *Youngina capensis* [69]. Between the thickened margin of the choana and the midline ridge for the tooth row, the ventral surface of the vomer is concave. Just posterior to the midline contact of the vomers is an anteroposteriorly short, ventral ridge. The ventral surface of the vomer bears a robust, dorsoventrally thick buttress of bone anteriorly at the level of vomer midline contact (figure 16*b*). This buttress, the ‘anterior vomerine buttress’ of Gow [25], is also present in *Milleretta rubidgei* [25] and was proposed as a diagnostic feature of millerettids. The anteroventral surface of the vomerine buttress bears two foramina, the ‘vomerine apertures’ of Gow ([25]; figure 16*b*). The ventral surface of this buttress supports two, enlarged vomerine teeth (‘fangs’ of [25]). These enlarged vomerine teeth are present in other millerettids, although they are proportionally larger in *Milleretta rubidgei* [25]. In *Milleretta*, the enlarged vomerine teeth are approximately 25% the circumference of the marginal dentition, whereas the enlarged vomerine teeth in *Milleropsis* are closer to 10%. A vomerine buttress bearing two enlarged vomerine teeth is also present in another specimen of *Milleropsis pricei,* BP/1/4203 (XA Jenkins, personal observation). These vomerine teeth differ from the vomerine fangs present in early tetrapods, which are not on the midline of the vomers and are generally larger than the marginal dentition [92,188].

#### 4.3.2. Palatine

The palatines of *Milleropsis* are structurally complex and include a ventral process abutting the maxilla and an ascending process for dorsal contact with the prefrontal, similar to many ‘parareptiles’ and neodiapsids ([61]; figure 10*b*) and unlike the paired, sheet-like palatines of most other early amniotes. The single, left palatine preserved Individual II is bounded by the pterygoid medially, the ectopterygoid posteriorly, the maxilla laterally, the vomer anteriorly and the prefrontal anterodorsally. The ventral surface of the palatine is complex, possessing both ridges and strongly developed concavities (figure 15). A row of teeth on a ventral ridge (‘T2’ of [189], ‘p2’ of [69]) extends onto the palatine from the pterygoid (figure 15*a*).

The anterior part of the palatine is damaged and is present as several fragments in our reconstruction (figure 15). However, based on these fragments and the complete palatine of Individual I, the anteromedial process would have been a triangular sheet of bone, tapering between the pterygoid medially and the vomer laterally and overlapping the dorsal surface of the vomer. A ventral ridge for the palatal tooth row extends anterolaterally along the ventral surface of the palatine, continuing directly from the pterygoid (figure 15*a*). This ridge terminates just posterior to the choana, which forms a deep emargination in the anterior edge of the palatine. A well-developed concavity or pocket is present on the ventral surface of the palatine, posterior and medial to the choana.

The maxillary ramus of the palatine *Milleropsis* is a thickened, ventrolateral process that connects the main body of the palatine with the maxilla (figure 15*b*). It is anteroposteriorly elongated, nearly matching the length of the palatine, unlike the short maxillary ramus in taxa that possess an elongate, suborbital fenestra, such as the neodiapsids *Youngina capensis* [56] and *Claudiosaurus germaini* [57]. The lateral portion of the maxillary ramus of *Milleropsis* consists of two processes that bound the orbitonasal foramen dorsally and ventrally: a short, lateral process that overlies the dorsal surface of the alveolar shelf of the maxilla, and a ventral process that is laterally concave for reception of the alveolar shelf. The foramen orbitonasale opens laterally on the anterior surface of the maxillary ramus, just ventral to the ascending process (figure 10*a*).

The presence of a foramen orbitonasale was previously considered to be a synapomorphy of Parareptilia [5,11], although it is absent in mesosaurs (e.g. *Mesosaurus* [190]). However, we note that the occurrence of a foramen orbitonasale appears to be strictly correlated with the presence of an ascending process of the palatine and is present in non-parareptiles that possess this morphology. An ascending process of a palatine and a foramen orbitonasal are present in neodiapsids including *Youngina* (personal observation BP/1/2871), *Prolacerta* [88], rhynchosaurs (the ‘infraorbital canal’ of [21] and [191]) and the stem turtle *Proganochelys quenstedti* [192]. The lateral expansion of the ascending process in these non-parareptilian taxa canalizes the posterior portion of the alveolar canal, which is normally present as a groove on the dorsal surface of the maxilla or at the junction of the maxilla, lacrimal and palatine as in *Orovenator* [15].

The dorsal surface of the palatine of *Milleropsis* features a robust, anterodorsally ascending process that contacts the ventral process of the prefrontal anteriorly within the orbit (figure 10*b*). A prominent ascending process of the palatine is present in many Permian tetrapods described as possessing strong prefrontal–palatal contact [5], including pantylid recumbirostrans [193] and edaphosaurid synapsids [59], and stem reptiles including *Milleretta rubidgei* (BP/1/3822), early pareiasaurs (‘vertical flange’ of *Embrithosaurus schwarzi,* [194]), procolophonoids (‘buttress-like’ prefrontal-palatal contact in *Saurodektes,* [55]), and *Australothyris smithi,* in which it has been referred to as the ‘orbitonasale’ ridge [73]. The dorsal surface of the palatine of *Milleropsis* does not bear a transversely oriented ridge or sheet that contributes to the nasal septum. This other structure is present in captorhinids, and was referred to as the ‘orbitonasale’ ridge by Heaton [43]. In captorhinids, this ridge frames a dorsally facing pocket which was thought to contain the maxillary process of the paranasal cartilage of the nasal septum [43, fig. 24*b*], and is absent in *Orovenator* [15], *Milleropsis pricei* and all neodiapsids in which this region has been adequately described (e.g. *Youngina capensis* [69]).

The posterior region of the palatine overlaps the pterygoid and ectopterygoid dorsally. The posterolateral margin of the palatine is embayed, forming the anterior border of the suborbital foramen (figure 15), which is enclosed posterolaterally by the ectopterygoid and laterally by the maxilla, similar to the condition in araeoscelidians [135], *Orovenator* [15] and neodiapsids, which possess an anteroposteriorly elongate suborbital fenestra framed by the maxilla, pterygoid and ectopterygoid. This contrasts with the morphology in procolophonians (e.g. *Sauropareion anoplus* [178]) and the stem-turtle *Proganochelys quenstedti* [192], in which the maxilla does not contribute to the suborbital foramen. A suborbital opening is absent altogether in synapsids [59], protorothyridids [84], bolosaurids [61] and acleistorhinids [11].

#### 4.3.3. Ectopterygoid

A single, left ectopterygoid is preserved in Individual II (figure 15) The ectopterygoid of *Milleropsis* is the shortest bone of the palate and contacts the palatine anteriorly, the maxilla and jugal laterally and the transverse flange of the pterygoid posteromedially. It is a dorsoventrally thin bone, although it bears a moderate lateral expansion for contact with the medial surfaces of the maxilla and jugal. The ectopterygoid forms the posterior border of the suborbital foramen anterolaterally.

The subtemporal fenestra deeply emarginates the posterior border of the ectopterygoid. A ridge on the ventral surface extends from the medial border of the subtemporal fenestra onto the anteroventral surface of the transverse flange of the pterygoid (figure 15*a*). This ventral ridge laterally frames a dorsal concavity, an ectopterygoid pocket (figure 15). The ectopterygoid possesses a long posterolateral process extending posteriorly from its initial contact with the jugal similar to many early reptiles, including *Orovenator* [15], and neodiapsids such as *Youngina capensis* [56], *Planocephalosaurus robinsonae* [195] and *Mesosuchus browni* [117]. It also differs from most early tetrapods, the diadectomorph *Limnoscelis paludis* [48] and the bolosaurid *Belebey vegrandis* [61] in which the ectopterygoid is rectangular and lacks a lateral process contacting the jugal. The ventral surface of the ectopterygoid of *Milleropsis* has no dentition, as in captorhinids (e.g. *Labidosaurus hamatus* [149]), diadectomorphs (e.g. Tseajai campi [142]), and most early neoreptiles (*sensu* [15]) other than the acleistorhinid *Delorhynchus cifelli* [96]. This differs from many other early amniotes, including some synapsids (e.g. *Casea* or *Edaphosaurus* [59]), araeoscelidians [3] and *Orovenator* [15], which have teeth on the ventral surface of the ectopterygoid.

#### 4.3.4. Pterygoid

Four pterygoids are present in our tomography data of BP/1/720; a complete left and incomplete right element in Individual II (figure 2*d*), as well as both incomplete pterygoids in Individual VI that are missing most of the palatal ramus (figure 3*d*). The pterygoid of *Milleropsis* is an anteroposteriorly elongate element and is the longest bone in the palate, slightly longer than the parabasisphenoid. The pterygoids of *Milleropsis pricei* contact all palatal elements and connect the palate with the braincase, and thus are structurally complex (figure 15).

The palatal ramus of the pterygoid is the largest portion of the pterygoid of BP/1/720, and it thins mediolaterally from its posterior contact with the basipterygoid process to its anterior contact with the vomer. The palatal ramus bears two tooth rows, one medially that follows the medial margin of the pterygoid bordering the interpterygoid vacuity, and one laterally that extends anterolaterally onto the palatine. This is similar to the condition in most early amniotes including ophiacodontids [59], early neoreptiles [15] and neodiapsids other than *Claudiosaurus* [57]. The lack of denticle shagreen on the pterygoid of *Milleropsis* differs from the condition in some other stem or early amniotes, such as the early captorhinid *Euconcordia* [52], synapsids including edaphosaurids and caseids [59], and acleistorhinid stem reptiles [96]. The medial pterygoid tooth row of *Milleropsis* (‘T3’ of [189]) extends from the basicranial articulation posteriorly and extends onto the vomer anteriorly. This medial pterygoid tooth row (‘T3’) is raised on a well-developed ventral ridge and takes the form of a single, distinct row of dentition posteriorly and continues as pairs of teeth not disposed into distinct rows anteriorly from the level of the anterior margin of the suborbital foramen. The more lateral pterygoid tooth row (‘T2’ of [189]) extends anteromedially from the basicranial articulation onto the ventral surface of the palatine, increasing in both width and number of teeth anteriorly. This second row (‘T2’) is on a ventral convexity, not as well defined as the ventral ridge bearing ‘T3’.

A sinusoidal channel is visible on the dorsal surface of the pterygoid near its articulation with the epipterygoid (figure 15*c*). This channel arcs first anterolaterally from a longitudinal sulcus, presumably for the medial palatine ramus of the facial nerve (CN VII) and the inferior nasal artery [43]. It then arcs strongly medially, where it follows the medial margin of the palatal ramus for the rest of its length (figure 15*c*). This channel, at least anteriorly, would have marked the pathways of the medial palatal ramus of the facial nerve, the nasal ramus of the ophthalmic division of CN V, the small superior nasal artery and the medial ramus for the inferior nasal artery as described in *Captorhinus laticeps* by Heaton [43], although in *Milleropsis*, no foramen is present.

**Figure 17.**
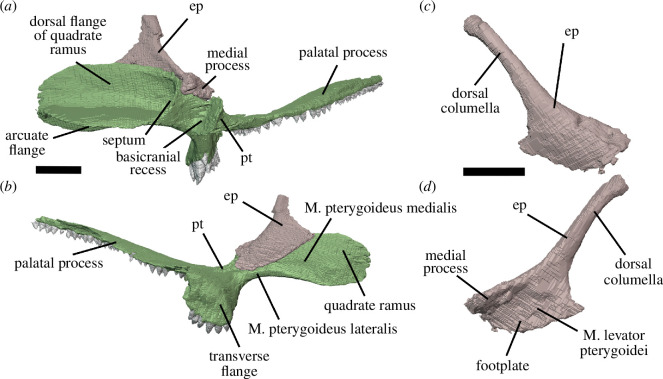
BP/1/720, holotype of *Milleropsis pricei*. Segmented basicranial region of Individual II and VI from µCT scans. (*a*) Left epipterygoid and pterygoid of Individual II in medial and (*b*) lateral view; (*c*) right epipterygoid of Individual VI in lateral and (*d*) medial views. Abbreviations: ep, epipterygoid and pt, pterygoid. Scale bar represents 2 mm.

The transverse flange of the pterygoid of *Milleropsis* is subrectangular, with lateral and posterior margins that form a weakly obtuse 100° angle with each other (figure 15). The transverse flange extends posteroventrally into the subtemporal fossa, extending ventral to the level of the maxillary tooth row (figures 15 and 17). A distinct lateral projection or transverse flange is considered a synapomorphy of Reptiliomorpha, being present in seymouriamorphs, diadectomorphs, some recumbirostrans and almost all amniotes [196,197]. In ventral view (figure 15*a*), the transverse flange of *Milleropsis* is oriented weakly anterolaterally, similar to the araeoscelidian *Araeoscelis gracilis* [62], procolophonians such as *Saurodektes* [55] and the neodiapsids *Claudiosaurus* [57] and *Coelurosauravus* [108]. This differs from the condition in most early amniotes, including the millerettid *Milleretta rubidgei* [25] and the early diverging neodiapsid *Youngina capensis* [69] in which the transverse flange is oriented primarily laterally [15] and from the anteriorly directed transverse flange of bolosaurids [61]. The transverse flange of *Milleropsis* bears a series of teeth (‘T1’ of [189]) on its posteroventral surface, located posterolaterally on a subtriangular platform of bone. The teeth of the transverse flange are the largest of the palate, nearly one-third the size of the marginal dentition.

The basicranial recess for articulation with the basipterygoid processes of the parabasisphenoid is located on the posteromedial surface of the pterygoid, posterior to the palatal ramus and medial to the transverse flange, taking the form of a subcircular and flat surface (figures 15 and 17) as in other stem reptiles [11] and most early synapsids ([59]; figure 17*a*). This stands in contrast to the medially directed basicranial recess of the lanthanosuchid *Lanthanosuchus* [198] and the acleistorhinid *Acleistorhinus* [11]. The dorsal surface of the pterygoid of *Milleropsis* bears a weak trough (as opposed to the fossa columella of squamates [199]) on the region joining the transverse flange and quadrate ramus lateral to the basicranial recess (figure 15*c*). A low ridge, possibly the orbitotemporal ridge of Heaton [43], is present lateral to the sulcus for the inferior nasal artery and marks the position of the orbitotemporal membrane that would have separated the orbit from the subtemporal fossa (figure 15*c*). The presence of this longitudinal sulcus and orbitotemporal ridge is similar to the condition described in *Captorhinus* [43] as well as that in the early reptile *Orovenator* (personal observation OMNH 74606) and the early diverging neodiapsid *Youngina capensis* (personal observation BP/1/2871). However, this region is poorly described in other early amniotes, precluding a more complete comparison.

The quadrate ramus of the pterygoid in *Milleropsis* extends posterolaterally from the basicranial recess, and its posterior end is laterally overlapped by the pterygoid ramus of the quadrate. The pterygoid does not make any contact with the squamosal, unlike in procolophonids and *Acleistorhinus* [11]. The quadrate ramus of *Milleropsis* is divided into two processes, the dorsal flange and the arcuate flange (figure 13*a*). The dorsal flange is mediolaterally thin, rising in dorsoventral height posteriorly, and bears a thickened ventral rim. In lateral view, the dorsal flange of the quadrate ramus bears a low crest or ridge along its ventralmost lateral surface, extending anteriorly from the pterygoid ramus of the quadrate to the posterior portion of the dorsal surface of the transverse flange. This crest probably marks the origination of the M. pterygoideus lateralis [43]. The origination of the M. pterygoideus medialis is probably present as an extremely shallow fossa on the lateral surface of the dorsal flange of the quadrate ramus immediately posteroventral to the epipterygoid footplate, although this is not well resolved in our segmentation.

A medial transverse shelf, the arcuate flange, extends from the ventral rim of the dorsal flange at an angle of approximately 100°, similar to *Orovenator* [15] and *Youngina* [69]. The arcuate flange is transversely broad posteriorly. An arcuate flange of the pterygoid is present in most early amniotes, including the early synapsid *Ophiacodon* [59] and the araeoscelidian *Araeoscelis gracilis* [62]. However, unlike in *Milleropsis*, the arcuate flange is developed mostly as a low keel in these taxa, as opposed to a medially directed flange. An arcuate flange is absent in non-amniotes such as *Seymouria baylorensis* [63] as well as recumbirostrans (e.g. *Rhynchonkos stovalli* [200]) and captorhinids, including *Captorhinus aguti* and *Euconcordia cunninghami* (K Jenkins 2022, personal communication), in which one had been previously described [52]. The dorsal and arcuate flanges of the pterygoid of *Milleropsis* are connected anteriorly by a vertical septum of bone that is concave posteriorly, forming a deep, subspherical concavity for the middle ear cavity (figure 17*a*). This vertical septum also forms a posterolateral buttress that braces the articulation of the basipterygoid processes with the basicranial recess posterolaterally (figure 17*a*).

#### 4.3.5. Epipterygoid

The left epipterygoid is preserved in Individual II (figure 17*a,b*) and the right epipterygoid is present in Individual VI (figure 17*c,d*), both remain in articulation with their respective pterygoids. The epipterygoid present in Individual II is missing the dorsal tip of the dorsal process, whereas the epipterygoid in Individual VI is complete. The epipterygoid of *Milleropsis* consists of a triangular base (‘footplate’), which lies on the pterygoid ventrally, and a mediolaterally thin, dorsal process that ends freely.

The anterior surface of the epipterygoid bears a medial process that dorsally ‘roofs’ the basal articulation but does not contribute to it (figure 17*a*). This condition is very similar to that of procolophonians (e.g. *Procolophon*; [64]), the ichthyosauriform *Chaohusaurus brevifemoralis* ([201], figure 4*d*) and neodiapsids (e.g. *Youngina* or *Prolacerta* [56]), which similarly possess a medial process of the epipterygoid that roofs, but does not contribute, to the basicranial articulation. This differs from the plesiomorphic condition within Tetrapoda in that the epipterygoid contributes at least partially (or entirely) to the basal articulation, as in temnospondyls (e.g. *Edops craigi* [91]), seymouriamorphs (e.g. *Seymouria baylorensis* [63]), captorhinids [202], synapsids (e.g. *Dimetrodon milleri* [59]), araeoscelidians (*Petrolacosaurus* [3]) and bolosaurids (e.g. *Belebey vegrandis* [61]). A medial process of the epipterygoid is absent in lepidosauromorphs (e.g. *Gephyrosaurus bridensis* [172]; *Diphydontosaurus avonis* [173]), in which the epipterygoid is located well posterior to the basicranial articulation and the skull. In *Milleropsis*, a small foramen pierces the epipterygoid slightly medial to the medial process. The medial surface of the epipterygoid is concave and, with a roughened portion of the quadrate ramus of the pterygoid, possibly marks the origination of the ‘M. levator pterygoidei homologue’ of ([43]; figure 17*a*).

The dorsal process of the epipterygoid of *Milleropsis* is oriented posterodorsally at an angle of 70–75° (figure 3*a*). The distal tip of the dorsal process is preserved in the right epipterygoid, and it is weakly mediolaterally expanded, forming a round, cup-like concavity that possibly supported a cartilaginous extension that continued to contact the parietals (figure 3*a*). However, the ossified portion of the epipterygoid ends freely. This differs from leptopleuronine procolophonids and the acleistorhinid *Feeserpeton oklahomensis*, in which the dorsal process of the epipterygoid is described as contacting the supraoccipital and/or prootic posterodorsally [112,176,203]. The anterior margin of the dorsal process is not heavily scarred nor keeled, in sharp contrast to *Captorhinus laticeps*, in which the dorsal process of the epipterygoid bears a ventrolaterally projecting ridge, which was thought to separate the origins of two divisions of the M. pseudotemporalis [43].

#### 4.3.6. Parabasisphenoid

**Figure 18.**
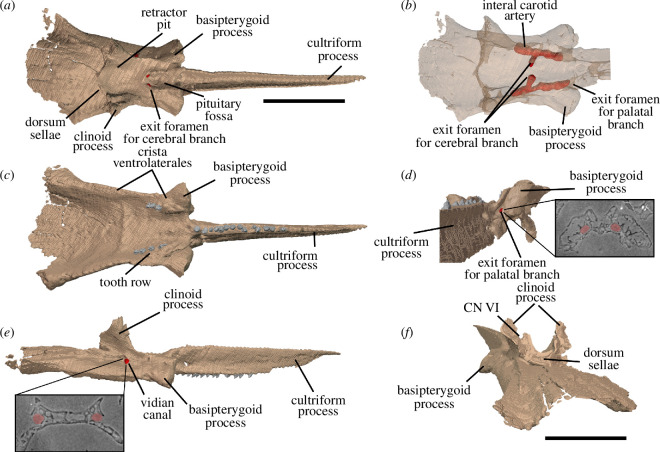
BP/1/720, holotype of *Milleropsis pricei*. Segmented parabasisphenoid and carotid arteries from µCT scans of Individual II. (*a*) Dorsal; (*b*) opaque dorsal; (*c*) ventral; (*d*) oblique anterolateral (vertically mirrored), (*e*) lateral; and (*f*) oblique posterodorsal views. Abbreviations: CN VI, abducens nerve foramen. Scale bar represents 3 mm.

The basisphenoid and parasphenoid of *Milleropsis* are indistinguishably fused and so described here as a single, compound element: the parabasisphenoid (figure 18). The parabasiphenoid consists primarily of four structures: the narrow and anteriorly extending cultriform process; the dorsal surface that contains the pituitary fossa, the clinoid processes and dorsum sellae; the anterolaterally facing basipterygoid processes; and the flat, ventral surface framed laterally by the crista ventrolaterales. Individual II possesses all these structures, and an additional parabasisphenoid is present in individual VI but is missing the cultriform process and the posterior portions of the crista ventrolaterales.

The cultriform process of the parabasisphenoid is elongate, extending from between the basipterygoid processes to well beyond the posterior end of the palatine (figure 15), nearly extending to the level of the internal nares as in many stem reptiles (e.g. *Orovenator* [15] *Youngina* [69]; and *Prolacerta* [56]). In contrast, the cultriform process is shorter or absent altogether in bolosaurids (e.g. *Belebey vegrandis* [61]) and procolophonians [86,204] often failing to extend anterior to the posterior border of the ectopterygoid. The cultriform process of *Milleropsis* remains horizontal throughout its length (figure 18*e*), lacking the anterodorsal slope (often more than 20°) present in captorhinids [109,156].

The cultriform process of *Milleropsis pricei* is dorsoventrally deep (figure 18*e*). Its dorsal bears two anteroposteriorly elongate laminae that together form a V-shaped trough. This trough is widest anterior to the pituitary fossae, attenuates anteriorly and probably supported an unossified sphenethmoid (figure 15*a*). An anteroposteriorly oriented ridge is present on the ventral surface of the cultriform process and bears at least 16 teeth. The presence of teeth on the ventral surface of the cultriform process is widespread among early amniotes, with notable losses occurring in bolosaurids [61] and in some members of the reptile crown group [7]. In *Milleropsis,* the rostral process of the parabasiphenoid is located immediately posterodorsal to the cultriform process and is a projection of bone that extends anteriorly between the basipterygoid processes. The anterior surface of the rostral process bears two projections, the crista trabeculares, which each bear a small oval facet on their anterior margin as in crown reptiles [205]. The crista trabeculares are slightly obscured in anterior view by the paired lamina of the cultriform process.

The basipterygoid processes are short projections of bone extending from the clinoid processes anteriorly to the level of the cultriform process (figure 15*d*). The basipterygoid processes have ovoid articular surfaces, each forming a single, anteriorly facing facet for the basicranial recess of the pterygoid. The short basipterygoid processes of *Milleropsis* are more similar to those of *Youngina capensis* [56] or *Procolophon trigoniceps* [64] than to other stem reptiles, including the putative early reptile *Hylonomus* [206], the araeoscelidian *Petrolacosaurus* ([3], figure 7) and *Orovenator* ([15], figure 12), in which the basipterygoid processes are laterally directed, elongate and finger-like projections in dorsal view.

**Figure 19.**
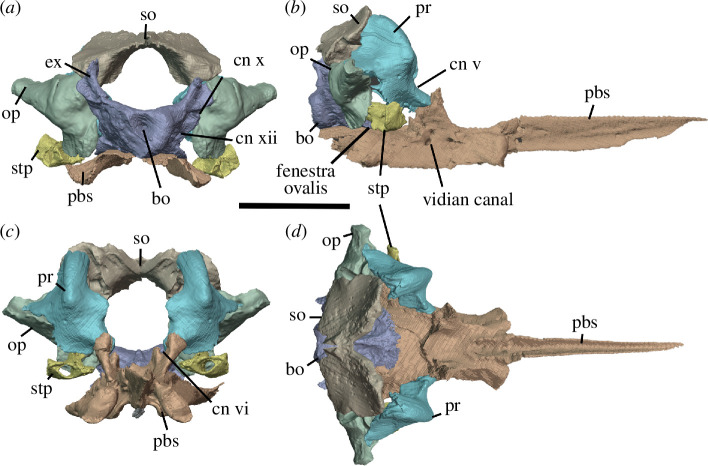
BP/1/720, holotype of *Milleropsis pricei*. Segmented braincase from µCT scans of Individual II in: (*a*) occipital view; (*b*) lateral view; (*c*) anterior view; and (*d*) dorsal view. Abbreviations: bo, basioccipital; cn v, trigeminal nerve foramen; cn vi, abducens nerve foramen; cn xii, hypoglossal nerve foramen; ex, exoccipital; op, opisthotic; pbs, parabasiphenoid; pr, prootic; so, supraoccipital; and stp, stapes. Scale bar represents 5 mm.

The dorsal surface of the rostral process of the parabasiphenoid bears an ovoid depression slightly posterior to the crista trabeculae, which forms the pituitary (hypophyseal) fossa (figure 18*a*). Anteriorly, the pituitary fossa opens into the cultriform recess. The margins of the fossa are framed by two low ridges. The pituitary fossa of *Milleropsis*, which houses the pituitary gland, is unusually elongate in BP/1/720. A shallow but mediolaterally broad fossa for the retractor bulbi muscle, the retractor pit, extends from the pituitary fossa anteriorly to a vertical sheet of bone, the dorsum sellae, posteriorly (figure 18). The fossa for the retractor bulbi broadly separates the dorsum sellae from the pituitary fossae, similar to the condition in *Youngina* [69] and *Orovenator* [15], although this structure is not reconstructed or noted in many comparative descriptions. This is unlike the condition in captorhinids (e.g. *C. laticeps* [43]) and procolophonians (e.g. *Macroleter* [175]), in which these two structures are nearly abutting. The dorsum sellae of *Milleropsis* is dorsoventrally low, but the parabasisphenoid is the primary contributor to this feature, as in most reptiles [43] and in varanopid synapsids (e.g. *Aerosaurus* [44]). This contrasts with some eupelycosaurs in which the prootic is the sole contributor to the dorsum sellae (e.g. *Dimetrodon milleri* [59]). The dorsum sellae of *Milleropsis* is framed by the clinoid processes laterally, which articulate with the ventral process of the prootic dorsally (figure 19). The dorsal tip of the clinoid process bifurcates distally, framing the canal for the abducens nerve (CN VI), and probably continued dorsally in cartilage as the pila antotica [207]. The position of CN VI lateral to the dorsum sellae is similar to the condition in the neodiapsid *Youngina* [208] and early saurians (e.g. *Prolacerta* [209]), but differs from the condition in synapsids (e.g. *Dimetrodon* [91]), traditional ‘eureptiles’ such as captorhinids (e.g. *Captorhinus* [43]) and *Petrolacosaurus* [4], in which CN VI extends through the dorsum sellae.

The ventral surface of the parabasisphenoid of *Milleropsis* is smooth and concave between the crista ventrolaterales (figure 15*c*). This concavity is more pronounced anteriorly near the basipterygoid processes; posteriorly, the ventral surface of the parabasiphenoid flattens. The ventral surface of the crista ventrolaterales of Individual II bears an anteromedially oriented row of four (on the left) or five (on the right) teeth anteriorly. These teeth do not merge onto the cultriform process, similar to the condition in other millerettids [25], including *Broomia* [86]. This differs from the condition in many other early amniotes in which most of the ventral surface of the parabasisphenoid is covered in denticles, such as the putative stem reptile *Hylonomus lyelli* [206], captorhinids (e.g. *Euconcordia* [52]), the acleistorhinid *Delorhynchus* [96], varanodontines (e.g. *Aerosaurus wellesi* [44]) and caseid synapsids (e.g. *Euromycter rutena* [168]).

The vidian sulcus of *Milleropsis* is entirely enclosed within the wall of the parabasiphenoid, forming a vidian canal. A ventrally inclined crest forms the lateral wall of the canal, a feature possibly plesiomorphic to Neoreptilia [15]. The entry foramen for the vidian canal is best observed in lateral view of Individual II (figure 18*e*), although its course can also be traced in Individual VI (figure 3*d*). This foramen is large and is located anteroventral to the clinoid processes. Tracing this canal anteriorly reveals the pathway of both the cerebral and palatal branches of the carotid artery. The cerebral and palatal arteries branch within the lateral wall of the braincase in *Milleropsis* (figure 16*b,d*), identical to the morphology present in procolophonians [210], the possible ‘neodiapsid’ *Lanthanolania* [102] and squamates, but unlike the condition in most early amniotes in which this branching occurs outside of the parabasisphenoid [211]. In *Milleropsis,* the cerebral branch of the carotid artery branches off from the palatal branch dorsolaterally, from where it exits the parabasisphenoid dorsally through the posterior portion of the pituitary fossa (figure 18*b*). The palatal branch continues anteriorly through the parabasiphenoid, exiting in a foramen medial to the basipterygoid process, as in *Lanthanolania* ([102]; figure 16*d*).

The parabasisphenoid contributes to the posteroventral margin of the fenestra ovalis, as in many early tetrapods and stem reptiles [212] including the neodiapsid *Youngina* [208], but unlike members of the reptile crown group, Sauria (e.g. *Prolacerta* [56,213]).

#### 4.3.7. Basioccipital

**Figure 20.**
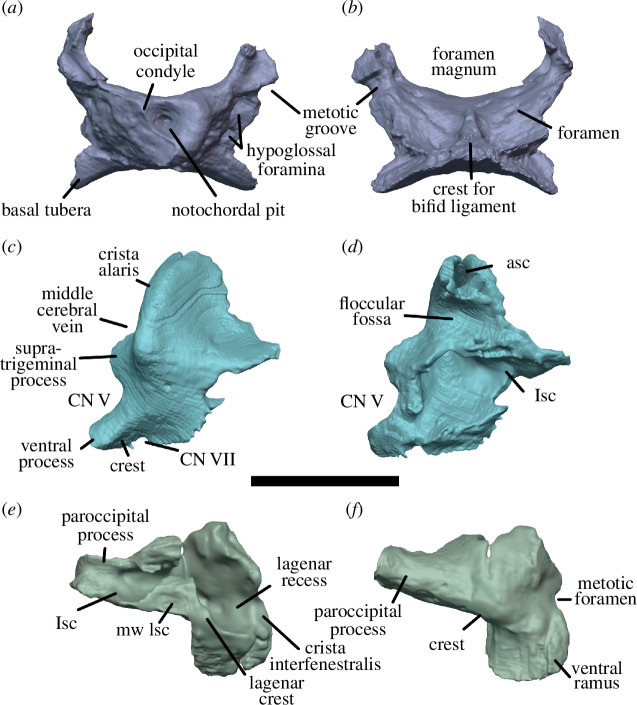
BP/1/720, holotype of *Milleropsis pricei*. Segmented basioccipital and left otic capsules from µCT scans of Individual II. (*a*) Basioccipital in posterior and (*b*) anterior views; left prootic in (*c*) lateral and (*d*) medial views; and opisthotic in (*e*) anterior and (*f*) posterior views. Abbreviations: asc, anterior semicircular canal; CN V, trigeminal nerve foramen CN VII, facial nerve foramen; lsc, lateral semicircular canal. Scale bar represents 3 mm.

Only a single basioccipital is preserved in BP/1/720, belonging to Individual II. It consists of a dorsoventrally thin anterior lamina that overlaps the parabasisphenoid dorsally and a posterior surface that is indistinguishably fused to the paired exoccipitals, forming the posteriorly directed occipital condyle and the ventrolateral margins of the foramen magnum (figures 19 and 20*e,f*).

The thin anterior lamina of the basioccipital overlaps the parabasisphenoid dorsally, as in most early amniotes, but unlike synapsids [50], pareiasaurs [175,194] and many archosauromorphs [60] in which the basioccipital and parabasisphenoids share a tightly interdigitated suture or are indistinguishably fused. There is no ‘fissure’ between these two elements in *Milleropsis*, or any other millerettid [25], contra statements by Laurin & Reisz [5]. In *Milleropsis*, the ventral surface of the anterior lamina is smoothly concave, except for weakly developed, paired and triangular ventrolateral projections, the basal tubera, which probably mark the insertion of epaxial musculature. The basal tubera are small compared with those of procolophonians, in which the tubera project ventrally well below the basioccipital condyle [64]. The basioccipital of *Milleropsis* does not form the posteroventral margin of the fenestra ovalis, unlike most Palaeozoic stem or early amniotes (e.g. *Captorhinus* [43]), but similar to some neodiapsids including Sauria [208].

The dorsal surface of the basioccipital bears a sagittal ridge anterior to the foramen magnum, mediolaterally thickened at its midlength, presumably for attachment of the bifid ligament of the medulla ([214]; figure 19*d*). The sagittal ridge is flanked laterally by two circular excavations, which are then framed by two, weakly developed posterolateral ridges. A small foramen pierces the anterodorsal surface of these ridges (figure 20*f*), which possibly represents a pneumatic foramen similar to that of neodiapsids including *Youngina* ([208], figure 3) and the rhynchosaur *Mesosuchus*, where topologically similar foramina have been interpreted as entrance foramina for the pharyngotympanic tubes [215]. However, *Milleropsis*, as in *Youngina* (personal observation AMNH 5561), lacks pneumatic sinuses within the basioccipital.

The occipital condyle of *Milleropsis* possesses a deeply concave posterior surface; the notochordal pit (figure 19*a*). A subcircular notochordal pit is also present in most early amniotes, including varanopid synapsids (e.g. *Varanops brevirostris* [155]), araeoscelidians (e.g. *Petrolacosaurus* [3]), early neodiapsids (e.g. *Youngina capensis* [56] and lepidosauromorphs [195]). This differs from archosauriformes, in which the occipital condyle is entirely convex, lacking any indication of a notochordal pit posteriorly [60]. The basioccipital of *Milleropsis* is fused to the exoccipitals dorsolaterally, although it is likely that these elements were separate during earlier ontogeny, as in *Milleretta rubidgei* [25].

#### 4.3.8. Exoccipital

As described above, the exoccipital is indistinguishably fused to the basioccipital, except for a slightly raised ventromedial ridge that appears to mark the ventral extent of the fused exoccipital (figure 20*e*). The following description assumes that the exoccipitals are similar in morphology and topology to the distinct exoccipital present in juvenile and subadult specimens of *Milleretta rubidgei* (e.g. BP/1/3818 and BP/1/3822), which also fuse with the basioccipital during later ontogeny (e.g. in RC 14, the holotype of *Milleretta rubidgei,* [29]). The exoccipitals of *Milleropsis* are lunate, bearing a dorsoventrally concave medial margin for the foramen magnum, and contact the supraoccipital dorsally, the opisthotic laterally and the basioccipital ventrally (figure 19*a*).

The exoccipitals are overlapped dorsally by the supraoccipitals, which make contact with the exoccipitals on the margin of the foramen magnum. In some procolophonids, including *Eomurruna yurrgensis* [107] and *Tichvinskia vjatkensis* (Ivakhnenko [216]), there is no osseous contact between these two elements, even in mature specimens. We cannot unequivocally state whether the exoccipitals of *Milleropsis* contacted each other ventrally at the midline or not due to fusion with the basioccipital.

The exoccipitals of *Milleropsis* are pierced by a large foramen anterolaterally, the lateral margin of which is framed by the opisthotic. This is the opening for the undivided metotic canal, which conveys cranial nerves IX–XI, as noted by Watson ([20]; figure 19*a*). The metotic foramen of *Milleropsis* is dorsoventrally enlarged, nearly a third of the mediolateral width of the head of the exoccipital wing (figure 20*f*), similar in size to the metotic foramen of *Youngina capensis* ([208], figure 4) but much larger than that of *Captorhinus* [43], in which the metotic foramen is approximately one-fifth the height of the exoccipital wing. The large metotic foramen of *Youngina* has been hypothesized to act as a pressure relief mechanism for the middle ear [217], and it is likely that the enlarged metotic foramen of *Milleropsis* served a similar role (figure 20). In anterior view, a dorsomedially inclined groove marks the pathway of the nerves associated with the metotic foramen (figure 20). The paired openings for the hypoglossal nerve (CN XII) open posteroventrally and are located posterior to the metotic foramen. In most early amniotes, the opening for CN XII is posterior to the metotic foramen, as in *Milleropsis*. However, in later branching procolophonids the opening for CN XII is anteriorly displaced, level with or anterior to the metotic foramen [107]. The paired foramina for CN XII are also visible in anterior view of the exoccipitals where they open blindly just anterior to the foramen magnum.

#### 4.3.9. Prootic

**Figure 21.**
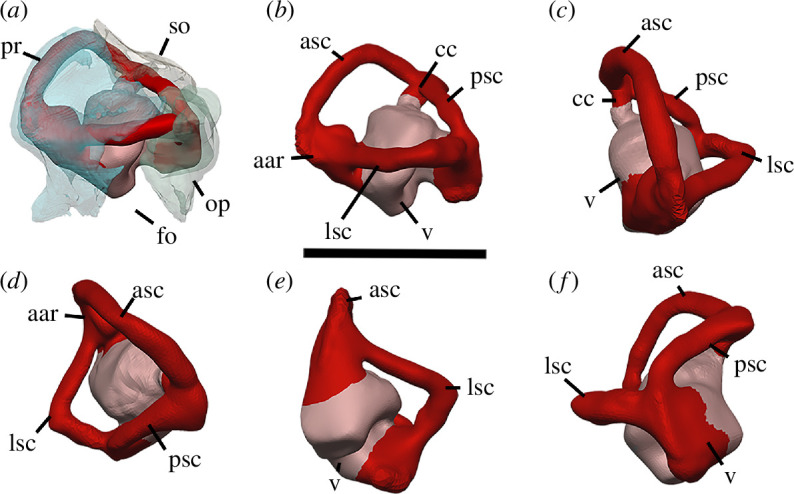
BP/1/720, holotype of *Milleropsis pricei*. Segmented left otic capsule and reconstructed inner ear of Individual II. (*a*) Opaque lateral view of otic capsule; (*b*) lateral view of inner ear; (*c*) anterior view of inner ear; (*d*) dorsal view of inner ear; (*e*) ventral view of inner ear; and (*f*) posterior view of inner ear. Red indicates features contained osseously, light pink indicates unossified portions of endosseous labyrinth. Abbreviations: aar, anterior ampullary recess; asc, anterior semicircular canal; cc, crus commune; fo, fenestra ovalisi; lsc, lateral semicircular canal; op, opisthotic; pr, prootic; psc, posterior semicircular canal; so, supraoccipital; and v, vestibule. Scale bar represents 5 mm.

Individual II possesses both prootics. The following description is based mostly on the more complete left element (figure 20*a,b*). The prootic of *Milleropsis* forms much of the lateral wall of the braincase and consists of three portions: a dorsally directed crista alaris (‘alar process’ of [43]; ‘crista alaris’ of [176]), a laterally directed contribution to the paroccipital process and a ventral process that articulates with the parabasiphenoid (figure 19*b*). The medial portion of the prootic is mostly unossified, unlike the prootics of pareiasaurs or archosauriforms in which the medial portion is ossified [60,106]. The prootic of *Milleropsis* also contributes to the margins of the anterior and lateral semicircular canals (figure 21).

The anterior surface of the prootic is marked by an anterolaterally inclined, bulging ridge: the crista alaris. The crista alaris marks the pathway of the anterior semicircular canal (ASC) on the lateral surface of the prootic, and it is concave medially for the ASC and the anterior ampullary recess (figure 19*d*). Just medial to the crista alaris is a crest, the supratrigeminal process, that forms the dorsal margin of the trigeminal notch for the trigeminal nerve (CN V; figure 19*b*). The supratrigeminal notch for the middle cerebral brain of *Milleropsis pricei*, and indeed most traditional stem reptiles (e.g. *Captorhinus* [43]; *Saurodektes kitchingorum* BP/1/3819), is located anterodorsally to the trigeminal notch, unlike the condition in early synapsids, including edaphosaurids (e.g. *Edaphosaurus boanerges* [115]) and sphenacodontids (*Dimetrodon* [59, plate 6]) in which the supratrigeminal notch is located more posterodorsally. A groove along the dorsal surface of the supratrigeminal notch probably marks the division of the medial cerebral vein from the main trunk of CN V. The floccular fossa is located posteromedial to the supratrigeminal notch and ventromedial to the crista alaris for the ASC (figure 20*b*). The floccular fossa bears no contribution by the supraoccipital.

An anteroventrally directed process, the ventral process of the prootic, extends anteriorly from the region of the trigeminal notch and approaches the clinoid processes of the parabasiphenoid ventrally (figure 19*c*). The anteroventral process of the prootic frames or encloses the CN V in crown reptiles ([7], character 74), differing from the condition in *Milleropsis* and most other non-saurians in which only the posterior margin of CN V is enclosed, as documented in captorhinids [43], araeoscelidians [3] and the neodiapsid *Youngina capensis* [208]. The ventral border of the ventral process bears a notch for the facial nerve (CN VII), a feature shared with *Milleretta* ([25], figure 9). This differs from most early amniotes or stem amniotes in which this is observable, including: recumbirostrans (e.g. *Brachydectes newberryi* [158], figure 6), synapsids (e.g. *Ophiacodon* [59, plate 2]), procolophonians (e.g. *Deltavjatia vjatkensis* [175], figure 19*b*), in which the facial nerve is completely enclosed by the prootic (i.e. expressed as a foramen), and is possibly autapomorphic for Millerettidae. An unossified gap *sensu* Evans [213] is present between the prootic and parabasisphenoid.

The prootic of *Milleropsis* bears a posterolaterally tapering lamina that covers the anteromedial surface of the paroccipital process of the opisthotic and indicates the pathway of the lateral semicircular canal (LSC). This prootic contribution to the paroccipital process is often referred to as the ‘paroccipital process of the prootic’ a feature well documented in neodiapsids (e.g. *Youngina* [208]), although it may also be widespread among crownward stem amniotes (e.g. *Captorhinus*; personal observation OMNH 44816) and crown amniotes such as synapsids (e.g. *Dimetrodon* [59, plate 14]). This lamina forms a small shelf that overhangs and contributes to the anterior margin of the stapedial recess. Farther ventrally, the prootic contributes to the anterior margin of a well-defined fenestra ovalis that is similar in shape and size to the footplate of the stapes (figure 19*b*). In Individual II, the left prootic contacts the quadrate, although this is probably artificial and due to the disarticulation of the braincase.

#### 4.3.10. Opisthotic

The opisthotic of *Milleropsis* is known from both elements preserved in Individual II (figure 13) and those described in Individual I by Watson [20]. The opisthotic of *Milleropsis* is a ‘y-shaped’ bone that forms the main component of the paroccipital process, the posterior portion of the inner ear cavity (figure 19) and the ventral margin of the posttemporal fenestra. The opisthotic consists of the laterally directed paroccipital process, a dorsal expansion that contacts the fused basioccipital-exoccipitals medially and the supraoccipital dorsomedially, and an expanded, plate-like ventral ramus that makes contact with the footplate of the stapes. The opisthotic forms the posterior and ventral portions of the fenestra ovalis and, together with the exoccipital, forms the metotic foramen for cranial nerves IX–XI (figure 20).

**Figure 22.**
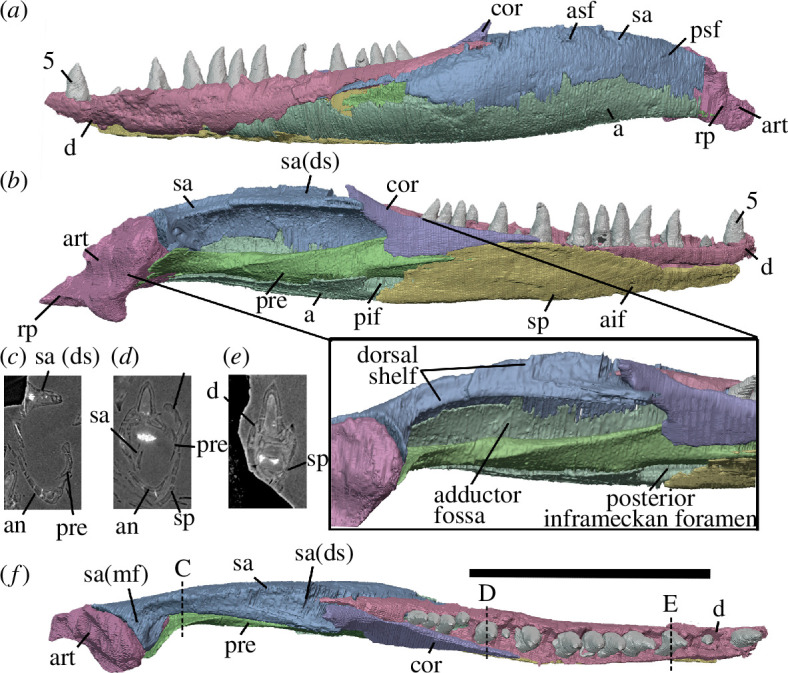
BP/1/720, holotype of *Milleropsis pricei*. Segmented left mandible from µCT scans of Individual II. (*a*) Lateral view of Individual II; (*b*) medial view of Individual II; (*c–e*) cross sections of left mandible of Individual II corresponding to dashed lines in (*f*), dorsal view of left mandible of Individual II. Abbreviations: a, angular; art, articular; aif, anterior inframeckelian foramen; asf, anterior surangular foramen; cor, coronoid; d, dentary; for, foramina; sa, surangular; sa(ds), dorsal shelf of surangular; sa(mf), medial flange of surangular; pif, posterior inframeckelian foramen; psf, posterior surangular foramen; pre, prearticular; sp, splenial; 1, first tooth position; and 5, fifth tooth position. Scale bar represents 1 cm.

The paroccipital process of the opisthotic of *Milleropsis* is robust and rodlike, extending laterally where it would have contacted the dorsomedial surface of the quadrate and probably the squamosal or tabular dorsally, in agreement with Gow ([25], figure 20). In Individual II, the paroccipital process ends slightly short of the quadrate, although this is probably an artefact of the braincase being slightly displaced in this region. The paroccipital process of Individual I has been described by both Watson [20] and Gow [25] as contacting the quadrate, and this observation is confirmed in Individual VI, where the occiput remains in articulation. The paroccipital process encloses much of the posterior semicircular canal as well as its ampullary fossa (figure 21*a*). The posterior semicircular canal is separated from the vestibule by a strongly developed medial wall, which nearly canalizes the lateral semicircular canal near the ampullary fossa for this canal (figure 20*b*). A crest runs mediolaterally on the posteroventral portion of the paroccipital process, causing the process to be convex in posterior view, although this crest is restricted to the paroccipital process and does not extend onto the ventral ramus, unlike in *Milleretta* (Jenkins *et al*. [38], in review). The paroccipital process of the opisthotic in *Milleropsis* is overlapped anteriorly by a short ‘paroccipital process’ of the prootic (figure 19*c*). The dorsal surface of the paroccipital process is smooth and featureless, except for where it articulates with the supraoccipital dorsally. At this contact, the opisthotic is dorsally swollen and bears an opening that marks the entrance of the posterior semicircular canal, which runs ventromedially from the supraoccipital. As preserved, the suture between the opisthotic and supraoccipital appears to be abutting, unlike the strong suture or fusion between these elements present in captorhinids [43], early synapsids [115], varanodontines [44] and archosauromorph reptiles ([218], figure 22).

At the junction between the paroccipital process and ventral ramus of the opisthotic is the articulation of the opisthotic with the fused exoccipital–basioccipital complex. A large opening, the metotic foramen, is present between the opisthotic and exoccipitals for cranial nerves IX–XI (figure 19*a*). The opisthotic is weakly concave for the passage of this foramen. The ventral ramus of the opisthotic in *Milleropsis* is plate-like and is moderately expanded distally. This is similar to the morphology present in many early amniotes (e.g. *Cotylorhynchus romeri* [59]; *Captorhinus laticeps* [43]), but is unlike the ventrally thinning ventral ramus of neodiapsids (e.g. *Youngina* [60]). The ventral ramus of the opisthotic of *Milleropsis* forms the posteroventral margin of the foramen ovale in which the enlarged footplate of the stapes rests (figure 20*c,d*). A dorsally sloping crest, the crista interfenestralis (or ‘intervestibularis’, *sensu* [219]) separates the metotic fossa from the vestibule ([43]; figure 20). This is similar to the condition in stem amniotes such as *Limnoscelis* [220], microsaurs and captorhinids [219], as well as the procolophonid *Leptopleuron* [176]. The crista interfenestralis also serves as a barrier between the metotic fossa and vestibule in synapsids such as ophiacodontids [59] and varanopids (e.g. *Aerosaurus*, personal observation UCMP V 35762 and *Mycterosaurus*, FMNH UC 692). A ‘lagenar crest’ *sensu* Heaton [43] separates the lagena from the scalae tympani (figure 20). The ventral end of the ventral ramus of the opisthotic is flat or convex, lacking an otic trough (*sensu* [137]) that leads to the fenestra ovalis, a feature documented in diadectomorphs, early diverging synapsids and reportedly in mesosaurid stem reptiles ([124]; figure 20*d*). There is no indication of a perilymphatic foramen along the medial surface of the ventral ramus.

#### 4.3.11. Supraoccipital

The supraoccipital of Individual II is weathered, such that much of the dorsal surface is missing lateral to the foramen magnum on the right side (figure 2*c*). Furthermore, the supraoccipital is rotated about 20° counterclockwise from its life position (figure 2*c*). The following description is based on this single element present in the tomography data and supplemented by information from the acid prepared and complete supraoccipital of Individual I. The supraoccipital of *Milleropsis* is a single, plate-like bone forming the dorsal portion of the foramen magnum and the medial margins of the posttemporal fenestrae (figure 13). It contacts the postparietals dorsally, the exoccipitals ventromedially, the prootic anteroventrally and the opisthotic posteroventrally.

The ventral surface of the supraoccipital is marked by a boomerang-shaped canal for the anterior and posterior semicircular canals (figures 19*b* and 21*a*). At the junction of these two canals, the supraoccipital is ventrally thickened, containing a fossa in which the ossified portion of the superior utriculus, or common crus, was enclosed. The supraoccipital lacks paired endolymphatic fossae, in contrast to stem amniotes [221] and the pareiasauromorph *Emeroleter* [222].

The posterodorsal surface of the supraoccipital is marked by a midline ridge, more developed than the weakly developed ridge present in *Milleretta* (BP/1/3818). The supraoccipital of *Milleropsis* lacks a median ascending process that divides the postparietals in posterior view, similar to the condition in procolophonians (e.g. *Eomurruna* [107]) and neodiapsids (e.g. *Youngina* [208]), but differing from some recumbirostrans (e.g. *Rhynchonkos* [200]) and captorhinids [156] in which the supraoccipital separates and broadly underlies the skull roof anteriorly. The supraoccipitals of *Milleropsis* and neoreptiles (*sensu* [15]) also lack the lateral ascending processes (*sensu* [219]), that are present in early or stem amniotes including captorhinids, recumbirostrans and the varanodontine *Aerosaurus* [44]. The supraoccipital of *Milleropsis* forms the medial border of relatively large posttemporal fenestrae laterally (figure 13), as described by Watson ([20], figure 19) unlike the ‘slit-like’ morphology of this fenestra reconstructed by Gow ([25], figure 20).

#### 4.3.12. Stapes

Both stapes are preserved in Individual II, and an additional right stapes was removed during acid preparation of Individual I [25]. The left stapes is rotated clockwise about 30° from its *in vivo* position (figure 1). The lateral surface was damaged during preparation to expose the paroccipital process of the opisthotic and the pterygoid wing of the quadrate, such that the stapedial shaft has a pocketed surface. Fortunately, the right stapes of Individual I preserves its lateral surface. The stapes of *Milleropsis* is a small but robust element, possessing a large footplate, stapedial foramen and dorsoventrally expanded shaft (figure 14).

The stapedial footplate is large and fits into the broad stapedial recess of the prootic anteromedially and the opisthotic posteromedially (figure 19). The medial surface of the footplate bears a deep, bowl-like concavity surrounded by a raised rim, as in *Milleretta rubidgei* [25]. As in most other tetrapods, the stapedial footplate of *Milleropsis* is not sutured or fused with the bones of the otic capsule (see [223] for review). This differs from captorhinids [43] and temnospondyls [91], in which the stapes is sutured to or fused to the otic capsule medially. The stapedial footplate of *Milleropsis* is relatively large, and is slightly shorter dorsoventrally than the ventral ramus of the opisthotic. The footplate, however, is still prominently reduced relative to other braincase elements when compared with many other early amniotes, including the synapsids *Edaphosaurus* [115] and *Ophiacodon* [59] or the captorhinid *C. laticeps*, in which the footplate is larger than the main body of the opisthotic [43]. In neodiapsids, the stapedial footplate is also reduced relative to the otic capsule, including the ventral ramus of the opisthotic (e.g. *Youngina* [208]). However, the stapes is still relatively robust. In *Youngina* [208]*,* the stapes appears slender due to an ossified ‘extracolumella’, although this ‘extracolumella’ may instead represent a break in the stapedial shaft. In the neodiapsid *Hovasaurus*, the stapes is similar in proportion to that of *Milleropsis*, lacking the slender, rodlike morphology present in later saurians [147]. Furthermore, the stapedial boss present on the medial surface of the quadrate in neodiapsids such as *Claudiosaurus* [57] and *Thadeosaurus* [224] may suggest the presence of a *Hovasaurus*-like stapes in these taxa, although the stapes is currently unknown in these neodiapsids.

In *Milleropsis*, a large stapedial foramen transmitting the stapedial artery pierces the shaft of the stapes at its midlength, similar to most Palaeozoic amniotes in which the stapes is known. This differs from most crown reptiles, which lack the stapedial foramen [60,154]), although a stapedial foramen is present in extant gekkotans [225], dibamids [26,226] and early in embryological development in amphisbaenians [227].

The stapedial shaft of *Milleropsis* is distally expanded, such that the distal end of the ossified stapes is taller than the stapedial footplate, similar to that of other millerettids such as *Milleretta* ([25], figure 10) and *Eunotosaurus* ([39], figure 2) and the varanopid *Mesenosaurus* [3]. In contrast, the stapedial shaft in other near and crown amniotes remains similar in height to the footplate (e.g. *Captorhinus laticeps* [43]) or is even more reduced (e.g. *Macroleter* [171]; *Youngina* [208]). Farther laterally, the stapes is supported by the stapedial process/boss of the quadrate (figure 14). In *Milleropsis*, an unossified extracolumella (or ‘tympanic process’ *sensu* [228]) probably extended laterally to contact the tympanic fossa shared between the quadrate, quadratojugal and squamosal as evident by the porous distal end of the stapes.

The dorsal surface of the stapes of *Milleropsis* is concave lateral to the stapedial footplate and therefore lacks a dorsal process (figure 14*a*). The presence of a dorsal process of the stapes is the plesiomorphic condition within Tetrapodomorpha, and is indeed present even in the early megalichthyid fish *Ectosteorhachis* [229] through the early branching members of Synapsida (e.g. ophiacodontids and caseasaurs [59]) and stem reptiles (e.g. captorhinids and araeoscelidians [5,20]), where it often articulates with the paroccipital process of the opisthotic dorsally. A dorsal process of the stapes has also been reported in acleistorhinid stem reptiles (e.g. *Acleistorhinus pteroticus* [41]) *Delorhynchus cifelli* [96]. However, in *Milleropsis* (as well as other millerettids [25]), procolophonians (e.g. *Deltavjatia* [175]) and neodiapsids (e.g. *Youngina capensis* [208]), the dorsal surface of the stapes is featureless.

#### 4.3.13. Sphenethmoid

An ossified ‘sphenethmoid’ or interorbital septum is not present in *Milleropsis* and is absent in other millerettids [25], similar to neodiapsids such as *Youngina* and crown reptiles [56]. Although possible non-saurian neodiapsid or lepidosauromorph *Elachistosuchus huenei* is reported as having a ‘sphenethmoid’ [230], this ossification is located within (and restricted to) the pila metoptica well posterior to the posterior nasal capsule and articulates with an ossification of the pila antotica posteriorly. This ‘sphenethmoid’ also lacks a midline interorbital septum ([230], figure 5*c*). The sphenethmoid of *Elachistosuchus* therefore non-homologous with the sphenethmoid of early amniotes, and possibly represents the orbitosphenoid seen in some crown reptiles. The absence of a sphenethmoid in millerettids and neodiapsids contrasts with the condition in most early amniotes, in which a ‘Y’-shaped sphenethmoid is formed by the interorbital cartilages, and is located between CN II posteriorly and the posterior nasal capsule anteriorly [231]. A sphenethmoid forming a distinct interorbital septum is known in stem and early crown amniotes, including captorhinids [43], synapsids [59] such as the mesenosaurine *Elliotsmithia* [232], araeoscelidians [3] and acleistorhinids (e.g. *Feeserpeton* [203]). The absence of ossified interorbital cartilages in *Milleropsis* and neodiapsids differs from the condition in stem-amniote recumbirostrans and procolophonian stem reptiles (e.g. *Procolophon*; [64]), which possess paired ossifications in this region [200].

#### 4.3.14. Endosseous labyrinth

The internal otic capsule of *Milleropsis pricei* is largely ossified in Individual II (figure 21*a*), except for the medial portion of the lateral semicircular canal and vestibule. The otic capsule is dorsoventrally compressed, more similar to neodiapsids such as *Youngina capensis* [208], the early turtle *Proganochelys quenstedi* [233] and the placodont *Placodus gigas* [234] than to the stem or early amniote *Captorhinus* [235], although reconstructions of the inner ear are lacking in most Carboniferous and Permian amniote clades, limiting the extent of this comparison. The degree of ossification of the semicircular canals in *Milleropsis* (and much of the braincase in general) is extensive compared with many tetrapods, such as the recumbirostran *Carrolla craddocki* [236] and *Captorhinus* [235], but is less ossified than that in synapsids (e.g. *Dimetrodon* [59]; varanodontines [237]), and pareiasaurs [106], in which the medial wall of the prootic is entirely ossified.

A portion of the common crus is ossified within the supraoccipital, where the anterior semicircular canal (ASC) and posterior semicircular canal (PSC) meet (figure 21*a*). The common crus is oriented posteriorly due to the greatly elevated ASC, although the ventral position of the common crus as well as its connection to the vestibule, could not be reconstructed due to the slight dislocation of the supraoccipital (figure 21*b*). The PSC is oriented posterolaterally from where it meets the ASC within the common crus, and is weakly arcuate (figure 21*f*). The course of the ASC is entirely ossified within the prootic and supraoccipital, and its path is strongly arcuate (figure 21*b,c*). The path of the LSC is largely straight, except for its posterior pathway within the opisthotic, in that it curves moderately posterolaterally, where it nearly meets the PSC within the lagenar recess. The ampullary fossa for the ampullae of the ASC and LSC is well developed, as is the ampullary fossa of the PSC within the opisthotic (figure 21*d*).

The ASC is the longest of the three canals, at 7.74 mm; the LSC is slightly shorter, at 7.58 mm, while the PSC is the shortest, at 6.77 mm. These measurements were taken in Dragonfly version 2022.2 using the ‘ruler’ tool and corresponded to the maximum curved length of the external surface of the semicircular canals. The angle between the ASC and the PSC is approximately 77°, that between the PSC and the LSC measures approximately 67° and between the LSC and ASC is approximately 85°.

The vestibule is subtriangular in lateral view and is contained mostly within the prootic and opisthotic, although its ventralmost extent was difficult to reconstruct due to lack of ossification in this region (figure 21*a*,*b*). The vestibule of *Milleropsis* appears intermediate between the short, rounded vestibule of captorhinids [235] and the narrower, more elongate vestibule present in *Youngina capensis* [208]. The lagenar recess is preserved in the ventral ramus of the opisthotic but is not well separated from the vestibule, and is not visible more than a weakly developed, posterior bulge in our reconstruction (figure 21*b*). However, there is no excavation on the dorsal surface of the lateral border of the parabasiphenoid or basioccipital that would suggest an elongate lagena.

### 4.4. Mandible

The lower jaw of *Milleropsis* is dorsoventrally slender, gradually increasing in height posteriorly (figure 22). The dentary and the angular contribute almost equally to the lateral surface of the jaw at its midlength, and the postdentary region of the lower jaw is relatively long compared with other millerettids, such as *Milleretta rubidgei* [25]. The adductor fossa opens primarily medially. The retroarticular process is well developed and is formed by the articular with some lateral support from the angular (figure 22*a*). In dorsal view, the lower jaw is weakly sinuous, matching the morphology of the maxillary tooth row.

#### 4.4.1. Dentary

**Figure 23.**
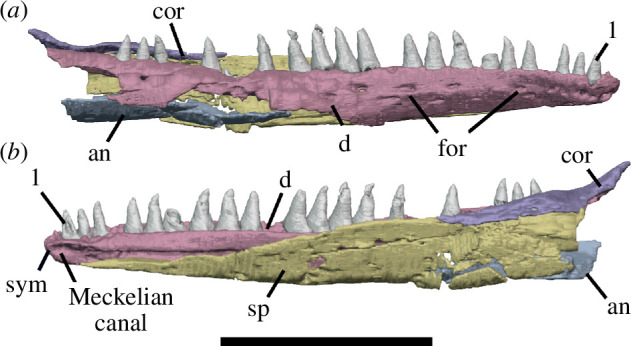
BP/1/720, holotype of *Milleropsis pricei*. Segmented right mandible from µCT scans of Individual IX. (*a*) Lateral view of Individual IX and (*b*) medial view of Individual IX. Abbreviations: an, angular; cor, coronoid; d, dentary; for, dentary foramina; sp, splenial, and sym, symphysis. Scale bar represents 5 mm.

Five dentaries are preserved in our scans of BP/1/720 (figure 1). The two dentaries preserved in Individual II were originally complete, although the symphyseal region was removed by Gow [25] to reveal the anatomy of the vomers (figure 2*d*). Fortunately, the symphyseal region is preserved in both dentaries present in Individual IX (figures 4*d* and 23). Other dentaries are preserved in referred specimens but are only visible in lateral view (figure 5*b,c*).

The dentary of *Milleropsis pricei* is anteroposteriorly elongate, forming much of the anterior portion of the mandible (figure 22). In lateral view, it contacts the angular ventrally from the 7th to 11th alveolus, the surangular ventrally from the 12th tooth position to its posterior tip, and the coronoid along its posteromedial surface (figures 22b and 23a). The dentary forms a longer contact with the surangular compared with the angular in lateral view, as in *Milleretta* ([25], figure 5). It forms the lateral surface of the mandible anteriorly, such that the splenial is not visible in lateral view (figure 22*a*). The lateral surface of the dentary in Individual IX is pierced by at least eight nutrient foramina, although an exact count cannot be determined due to damage to the lateral surface of most of the dentaries (figure 22*d*). The posterior end of the dentary overlaps the lateral surface of the surangular, attenuating posterodorsally and does not bifurcate in dorsal view.

A mediolaterally broad alveolar shelf forms the dorsal margin of the Meckelian canal. This shelf is formed by two anteroposteriorly oriented laminae on the dorsal surface of the dentary, the labial and lingual parapets of the marginal dentition (figure 9). The labial shelf is slightly taller than the lingual shelf, as present in many stem or early amniotes including some recumbirostrans (e.g. *Llistrofus* [180]), the early captorhinid *Euconcordia* [238], the owenettid *Ruhuharia* [239] and the neodiapsid *Youngina* [69]. This differs from the morphology in taxa with ‘pleurodont’ dental implantation such as weigeltisaurids (e.g. *Coelurosauravus* [97,108,213]), some lepidosauromorphs (e.g. *Marmoretta* [78]) and the neodiapsid *Claudiosaurus* [57], in which the lingual wall is absent or reduced. Between these two shelves is a concave surface for the marginal tooth row. The most complete dentary bears 23 alveoli, far more than the 13 teeth reported in the dentary of *Milleretta rubidgei* [25]. The dentary teeth are similar to the maxillary teeth in being conical and slightly recurved. The implantation can be classified as broadly subthecodont, with the dentary bearing shallow alveoli separated by low interdental plates (figures 8 and 9). Replacement crowns are evident in several tooth positions, positioned basal to the erupted tooth within the alveolus, similar to other millerettids (e.g. *Milleretta*; personal observation BP/1/3822). This contrasts with the lingual replacement crowns of other stem reptiles such as the neodiapsid *Youngina capensis* ([69]; figure 9*b–d*). The inferior alveolar canal for the inferior alveolar nerve runs ventral to the tooth row throughout the length of the dentary.

The dentary forms the dorsal and ventral margins of the anteriorly attenuating Meckelian canal, which splits the anterior tip of the dentary anteriorly such that the dorsal and ventral margins of the dentary are not in contact with each other at the mandibular symphysis (figure 23). The dentary of *Milleropsis* is the sole contributor to the mandibular symphysis, a feature shared with most stem reptiles including araeoscelidians (e.g. *Araeoscelis* [62]), procolophonids (e.g. *Sauroparieon* [178]), and early neodiapsids [69], as well as ophiacodontid synapsids [59]. This differs from early tetrapodomorphs (e.g. *Whatcheeria deltae* [240]), recumbirostrans [97], captorhinids (e.g. *Captorhinus laticeps* [43]) and pareiasaurs (e.g. *Deltavjatia* [175]) in which the splenial extends to the level of, and usually contributes to the mandibular symphysis. The anterior end of the dentary of *Milleropsis* is pierced by the Meckelian canal, as in other millerettids [25] and the neodiapsid *Youngina* [69]. The symphyseal facet of *Milleropsis* is located dorsal to the Meckelian canal.

#### 4.4.2. Angular

Three angulars are preserved in our scans of BP/1/720, of which the left angular of Individual II is nearly complete (figure 2*b*), although the suture between the angular and surangular was difficult to reconstruct due to preparation damage to the lateral surfaces of these bones. The angular of *Milleropsis* is the longest bone in the mandible and is the primary element forming the ventral margin of the lower jaw posteriorly (figure 22*a*). It contacts the dentary anteriorly, the splenial anteromedially, the surangular dorsolaterally, the prearticular dorsomedially, and the articular posteriorly (figure 22).

The lateral exposure of the angular decreases as it reduces in height anteriorly. At the level of the ninth alveolus, the angular is overlapped by the splenial ventrally, although it extends forward for some distance, ending in a point medial to the dentary and lateral to the splenial. The posterolateral surface of the angular is overlapped dorsally by the surangular. The angular bears a concavity on its dorsomedial margin at the junction between the angular, prearticular and splenial, which forms the ventral rim of the posterior inframeckelian foramen (foramen intermandibularis caudalis of [95]; figure 22*b*). Farther posteriorly, the angular laterally overlaps the articular but does not contribute to the retroarticular process (figure 22*b*). The angular forms the ventral surface of the mandible from the condylar region to the level of the tooth row and becomes overlapped by the splenial medially. The ventral surface of the angular bears a moderately developed keel, as in *Milleretta* (BP/1/3822).

#### 4.4.3. Surangular

Both surangulars are preserved in Individual II and an additional, fragmented surangular is present in Individual VI (figure 23). The surangular of *Milleropsis* is an elongate element, forming the dorsal portion of the posterolateral mandible as well as forming the dorsal margin of an adductor fossa. The dorsal surface is medially expanded and forms a dorsomedially facing shelf (figure 22). The surangular bears a cup-shaped medial flange for articulation with the articular posteriorly.

The surangular attenuates anterior to the level of the coronoid eminence, where it bears a concavity for articulation with the dentary (figure 22*f*). The dorsal surface of the surangular is mediolaterally expanded, forming a dorsally facing shelf (figure 22). A mediolateral expansion of the surangular is a synapomorphy of varanodontines amongst early synapsids [140,241]. However, it is also present among stem reptiles such as the owenettid *Saurodektes kitchingorum* (BP/1/4195), and the neodiapsids *Youngina capensis* [69], *Hovasaurus* [147] and weigeltisaurids [108]. The surangular of *Milleropsis* extends from the medial flange for the articular posteriorly to the level of the coronoid eminence, where it accommodates the posterodorsal process of the coronoid. The posterior end of the surangular of *Milleropsis* curves medially such that the medial margin is concave in dorsal view. The medial flange of the surangular that braces the articular is concave posteriorly (figure 22*c*). The surangular of *Milleropsis* terminates posteriorly at the level of the lateral condylar surface of the quadrate, sheathing the articular for approximately half of its length in lateral view. The posterior surangular foramen is located anterolateral to the glenoid fossa of the articular, transmitting the chorda tympani branch of CN VII, similar to the condition in *Milleretta* ([25], figure 5), and the archosauromorphs *Prolacerta* and *Proterosuchus* [79]. An anterior surangular foramen is present just posterior to the posteriormost extent of the dentary (figure 22).

#### 4.4.4. Splenial

A total of five splenials are preserved in the scanned individuals of BP/1/720. Similar to the dentaries, the anterior portions of the splenials of Individual II were removed by Gow [25] to reveal the palatal anatomy. The splenial of *Milleropsis* is a mediolaterally thin bone, forming most of the medial surface of the anterior mandible as well as the medial wall of the Meckelian canal (figure 22*b*). The dorsal portion of the splenial forms an overlapping suture with the dentary anteriorly and the coronoid posterodorsally. The splenial overlaps the prearticular posteriorly, the angular posteroventrally and the dentary ventrally. The splenial does not contribute to the mandible symphysis, instead ending at the level of the third dentary alveolus (figure 23).

The lateral surface of the splenial bears two laminae that join anteriorly. The first, dorsalmost lamina underlies and supports the alveolar shelf of the dentary. The second, most ventral lamina is overlapped by the dentary on the ventral surface of the mandible. The splenial possesses a foramen on its medial surface at the level of approximately the 13th alveolus. This is the anterior inframeckelian foramen (anterior mylohyoidal foramen (figure 22*b*)). A similar foramen is present on the medial surface of the splenial in most neoreptiles, including procolophonians (e.g. *Procolophon* [64]) and neodiapsids (e.g. *Youngina capensis* [69]), and has also been reported in *Orovenator* [15] and the acleistorhinid *Feeserpeton* [203]. In contrast, the medial surface of the splenial of stem and early amniotes, including the diadectomorph *Limnoscelis* [48], varanodontines (e.g. *Aerosaurus* [44]) and all early synapsids in which this is known [242], lack an anterior inframeckelian foramen, although damage to the medial surface of the splenial can easily obscure the presence of this feature.

Farther posteriorly, the splenial of *Milleropsis* contributes to the anterior margin of the posterior inframeckelian foramen, as in most stem reptiles excluding bolosaurids (e.g. *Belebey* [61]). The splenial in most ‘pelycosaur’ grade synapsids does not contribute to the posterior inframeckelian foramen (e.g. varanodontines 155), with *Edaphosaurus* being a notable exception [115]. There is no postsplenial, as in all other amniotes except *Petrolacosaurus* [3], although it is likely that this ‘postsplenial’ of this taxon is a fragment of the angular.

#### 4.4.5. Coronoid

The left and right coronoids are preserved in Individual II, and an additional, right coronoid is preserved in Individual VI (figures 22 and 23). The coronoid of *Milleropsis* is a dorsoventrally thin bone and forms the anterior margin of the adductor fossa (figure 22). The coronoid bears a long, anterior process, a posterodorsal process that forms a dorsoventrally low coronoid eminence, and a reduced posteroventral process (figure 22*b*). The coronoid contacts the dentary and splenial anteroventrally and the surangular posteroventrally.

The anterior process of the coronoid of *Milleropsis* attenuates anteriorly, ending at the level of approximately the 20th dentary alveolus (figure 22*b*). In contrast, the anterior process of the coronoid in later branching procolophonids is anteriorly short, in some cases not even extending to the level of the last tooth position, as in *Leptopleuron* [51] or *Eomurruna* [107]. The ventral margin of the anterior process of *Milleropsis* rests upon (and is supported by) a medially projecting flange on the prearticular ventrally and a medial ridge on the alveolar shelf of the dentary anteriorly. The entire medial surface of the coronoid is edentulous as in all known reptiles except for the putative early reptile *Hylonomus* [206] and acleistorhinid stem reptiles such as *Delorhynchus cifelli* [243] and *Feeserpeton oklahomensis* [103].

A posterodorsal process of the coronoid extends from the dorsal rim of the coronoid onto a groove on the surangular. This groove extends posteriorly a short distance farther than the posterodorsal process, suggesting that this process was broken distally. The ventral surface of the posterodorsal margin bears a ventral facet for this articulation. The coronoid eminence is extremely low, developed as a minor convexity between the coronoid, surangularand dentary. The coronoid of *Milleropsis* bears a poorly developed posteroventral process that does not contribute to the ventral margin of the adductor fossae (figure 22*b*), similar to mesenosaurine varanopids (e.g. *Mesenosaurus* [40]) and neodiapsids [57]. This differs from the condition in other early amniotes such as the caseid *Cotylorhynchus* [59], the acleistorhinid *Feeserpeton* ([203], figure 3*d*), and procolophonians such as *Procolophon trigoniceps* ([64], figure 13*b*) that possess a distinct posteroventral process that forms the anteroventral margin of the adductor fossae.

*Milleropsis* has a single coronoid ossification, similar to that of all neodiapsids (e.g. *Youngina* [69]) and crown reptiles (e.g. *Prolacerta* [56]). In contrast, earlier stem reptiles including the acleistorhinid *Delorhynchus cifelli* [243], the ‘diapsid’ of uncertain affinities *Maiothisavros dianeae* [244], and the araeoscelidians *Araeoscelis gracilis* [157] and *Zarcasaurus tanyderus* [177] have two coronoid ossifications. Almost all early synapsids also bear two coronoids (e.g. *Haptodus* [59,245]), whereas early tetrapods often possess three [246].

#### 4.4.6. Prearticular

Both prearticulars are well preserved in Individual II (figure 2), although no other prearticulars are present in other scanned individuals. The prearticular of *Milleropsis* is a complex element, forming the medial surface of the mandible posteriorly, the dorsal margin of the posterior Meckelian foramen and the ventromedial rim of the adductor fossa (figure 22*b*).

The anterior end of the prearticular underlies the coronoid, and the suture between these two elements is supported by a strongly developed ridge on the medial surface of the prearticular (figure 22*b*). This ridge dissipates posteriorly, and the medial surface of the prearticular dorsal to this ridge is inflected ventromedially. The rim of the adductor fossa is marked by another lamina, which travels through the length of the element. The medial surface of the prearticular is overlapped by the broad, sheet-like splenial anterior to the level of the posterior meckelian foramen and the prearticular forms the posterior margin of this foramen (figure 22*b*). The prearticular forms the ventral margin of the mandible together with the angular. The posterior end of the prearticular overlaps the articular medially. This region is strongly inclined dorsolaterally, almost twisted, overlapping the lateral surface of the articular and ventrally supporting the medial quadrate condyle (figure 22*b*).

#### 4.4.7. Articular

Three articulars are present in the scanned block, both elements from Individual II and an additional right articular preserved in Individual VI. The articular is an anteroposteriorly overall short element, consisting of a long, retroarticular process posteriorly, a short anterior process and a dorsal cotylar region (figure 22). The articular of *Milleropsis* is overlapped by the surangular anteriorly and laterally, the angular ventrolaterally and the prearticular medially.

The condylar region consists of two cotyles for articulation for the quadrate. Each cotyle bears a moderately developed ridge located medially on its surface (figure 22*f*). The lateral cotyle is elevated dorsally relative to the medial condyle and it is sloped anterodorsally by about 20°. The medial cotyle is located more ventrally and bears a strong, medial development that overhangs the prearticular below. The jaw articulation is approximately level with the mandibular tooth row, unlike in leptopleuronine procolophonids in which the cotyles are located quite ventrally to the mandibular tooth row [51,176]. A large retroarticular process extends posterior to the condylar region and is oriented primarily posterodorsally, possibly for the insertion of the pterygomandibularis musculature (figure 22*f*). The dorsomedial surface of the retroarticular process in *Milleropsis* is strongly concave, such that the retroarticular process appears hooked or upturned in lateral view, not unlike the upturned retroarticular process of some archosauromorphs ([60], figure 20).

#### 4.4.8. Hyoid apparatus

An ossified hyoid apparatus is commonly preserved in traditional ‘parareptiles’, where it has been described in *Mesosaurus tenuidens* [89], *Emeroleter* [166], pareiasaurs [111] and procolophonids [107]. In *Milleropsis*, the only preserved portions of the hyoid apparatus are the paired ceratohyals that are preserved in place. The elongate, tubular pair of first ceratohyals extend from the transverse flange of the pterygoid anteriorly to just posterior to the retroarticular process in both Individual II and BP/1/4203 (figure 2*d*) and are very similar in length to the hyoids of the early millerettid *Broomia* [12] and the varanopids *Elliotsmithia* [232] and *Heleosaurus* [73]. The elongation of the first ceratohyals, and specifically their posterior extension behind the skull was previously considered to be a synapomorphy of Archosauromorpha [60] and the Mesenosaurinae, however, Spindler *et al*. [241] noted their absence in most varanopids, in contrast to previous literature on the subject (e.g. [155]). The first ceratohyal of *Milleropsis* is pinched mediolaterally at its midlength and ends in an anteriorly facing, rounded concavity, lacking the expansion present in some procolophonians ([64]; figure 2*d*). No basihyal or medial copula is evident in the tomography data (figures 2*d* and 3*d*).

## 5. Discussion

### 5.1. *Milleropsis* shares derived anatomical features with Neodiapsida

Here, we observed that at least nine cranial features present in *Milleropsis* are shared with neodiapsids, including crown reptiles, including: exclusion of the lacrimal from the naris, loss of the occipital shelf of the squamosal, a stapedial boss of the quadrate, the presence of a tympanic fossa on the posterolateral surface of the skull, ectopterygoid bearing a posterolateral process, loss of an ossified sphenethmoid, loss of epipterygoid contribution to the basicranial recess, presence of the pathway of the abducens nerve (CN VI) through the clinoid process and loss of the dorsal process of the stapes. These features are absent in taxa that have traditionally been classified as early diverging ‘eureptiles', such as protorothyridids, captorhinids and araeoscelidians. The distributions of these features therefore invite a reconsideration of the phylogenetic affinities of *Milleropsis* and other millerettids, and argue for a more crownward position of millerettids among stem reptiles. In the following sections, we more closely examine the distribution of these features in stem reptiles.

#### 5.1.1. Exclusion of lacrimal from naris

An anteriorly elongate lacrimal that does not contribute to the posterodorsal margin of the external naris is considered a synapomorphy of Neodiapsida [5,7,19], that was also acquired, convergently, in procolophonian ankyramorphs [11]. Previous studies have interpreted millerettids, specifically *Milleretta* [30], as possessing an anteriorly long lacrimal that contributes to the external naris (e.g. [124]). Existing morphological descriptions of millerettids, including *Milleropsis* [20] and *Milleretta* [25], note that the anterior extent of the lacrimal is not determinable in specimens under description, although Carroll [100] reconstructed *Milleropsis* as lacking a lacrimal contribution to the narial margin. Our scans demonstrate that all specimens of *Milleropsis* lack a lacrimal contribution to the external naris due to an anterodorsally expanded maxilla that contacts the nasal in lateral view (figure 5). The lacrimal does not contribute to the external naris in *Milleretta* (personal observation R.C. 14, BP/1/3818 and BP/1/3822) or the hypothesized millerettid *Eunotosaurus* [45]. Early amniotes including captorhinids [43] and araeoscelidians [3] possess a broad contribution of the lacrimal to the external naris. Later branching stem reptiles such as *Orovenator* possess a smaller contribution [15], whereas procolophonoids [55], millerettids (this study) and neodiapsids [57] lack any such contribution.

#### 5.1.2. Occipital shelf of squamosal lost

In early amniotes (e.g. *Petrolacosaurus* [3]), the squamosal possesses an occipital shelf or flange that overlaps the dorsal process of the quadrate, obscuring the quadrate in lateral and occipital view [7]. This occipital shelf is nearly ubiquitous among early stem reptiles such as the acleistorhinid *Feeserpeton* [103], the mesosaurid *Mesosaurus* [89] and the bolosaurid *Eudibamus*. In *Milleropsis* and other millerettids, the squamosal lacks an occipital shelf, and the dorsal process of the quadrate is visible in lateral and occipital view (figure 14). The absence of an occipital shelf is a feature *Milleropsis* shares with neodiapsids (barring weigeltisaurids, in which the squamosal and parietals are modified into an ornamented frill [154]). The reduction and eventual loss of an occipital shelf of the squamosal in crownward stem reptiles has been linked with the development of a tympanic emargination on the posterior surface of the quadrate or in promoting mobility of the quadrate, allowing for the eventual evolution of streptostyly in Squamata [154].

#### 5.1.3. Stapedial boss of the quadrate

*Milleropsis* and other millerettids bear a raised shelf or boss on the medial surface of the quadrate that receives the distal (or quadrate) process of the stapes in a synovial joint. A similar stapedial boss has been documented in neodiapsids, being present in taxa including the early diverging *Youngina* and more crownward taxa such as *Claudiosaurus* [57] and *Acerosodontosaurus* (personal observation MNHN 1908-32-57). Our review of the suspensorium in stem reptiles reveals that a stapedial boss or shelf of the quadrate is a feature present in nearly all neoreptiles, but notably absent in other putative stem reptiles such as captorhinids [43] and araeoscelidians [157], which bear the stapedial recess present in all early amniotes and many reptiliomorphs [59,196]. A stapedial boss may have promoted mobility of the stapes and improved its function as an ear ossicle for sound transmission, allowing auditory cues to travel from the lateral skull through the middle ear more effectively than a stapes held rigidly within a stapedial recess [247].

#### 5.1.4. Tympanic fossa on the posterolateral surface of the skull

The presence of a tympanic fossa on the posterolateral surface of the skull has been a longstanding feature linking millerettids with crownward reptiles, although the structural architecture of the tympanum differs between these taxa [19,20,25]. In millerettids, the tympanic fossa is present on the quadrate, quadratojugal and squamosal, although it is mostly supported by the latter two elements. A somewhat similar tympanic fossa between the squamosal, quadratojugal has been documented in procolophonians, although in these taxa there is no contribution to this fossa by the quadrate, and this fossa extends onto the skull roof [171]. In the context of saurian evolution, the presence of a tympanum has been invariably codified based on the presence of an emargination or conch on the posterior surface of the quadrate (e.g. [60,154]). However, the tympanic membrane of extant saurians is also supported by the quadratojugal and squamosal [248–252]. Early archosauromorphs (e.g. *Prolacerta* [79]) and lepidosaurs (e.g. *Clevosaurus* [253]) possess a tympanic fossa on the quadrate, squamosal and quadratojugal. The posterolateral skull of many non-saurian neodiapsids possesses a distinct emargination. The squamosal is shallowly concave in the early diverging neodiapsid *Youngina* [56] and the possible tangasaurid *Acerosodontosaurus* [123], whereas the *Endothiodon* Zone younginiform (SAM-PK-K7710) and *Thadeosaurus colcanapi* [224] possess a tympanic emargination supported primarily by the quadrate with some contribution by the squamosal and quadratojugal. The quadrate is only one of many elements contributing to the external ear of extinct and extant reptiles, and future studies wishing to fully capture the variation of the tympanic emargination must include morphologies present on the squamosal and quadratojugal.

#### 5.1.5. Loss of dorsal process of stapes

Under traditional amniote phylogenies, the loss of the dorsal process of the stapes is considered a synapomorphy of neodiapsids (e.g. *Hovasaurus* [147]) relative to other ‘eureptiles’ [5]. However, the dorsal process of the stapes is also absent in *Milleropsis* and all other millerettids (figure 20), as shown here and repeatedly documented in the literature [20,25]. Nevertheless, the dorsal process is often scored as present in millerettids, in existing phylogenetic data matrices (e.g. [8]). In early amniotes, the dorsal process of the stapes serves as an additional brace between the braincase and suspensorium, where it is usually in contact with the ventral surface of the paroccipital process of the opisthotic, a feature exemplified in captorhinids [43] and araeoscelidians [157]. The loss of a dorsal process and its articulation with the braincase is linked to the reduced function of the stapes as a brace between the braincase and lateral skull in Neodiapsida, and improved function in auditory sensitivity [217].

#### 5.1.6. Ectopterygoid bearing a posterolateral process

*Milleropsis* possesses an elongated posterolateral process of the ectopterygoid that joins the palate with the jugal and maxilla and forms the anterior emargination of the subtemporal fenestra (figure 15). This feature is well documented in neodiapsid stem reptiles, for example, it is present in the stem reptile *Orovenator* ([15], figure 11*a*) and the early diverging neodiapsid *Youngina* ([69], figure 2*b*), as well as crown-group saurians such as rhynchocephalians (e.g. *Clevosaurus* [253], figure 14) and rhynchosaurs (e.g. *Mesosuchus*; personal observation SAM-PK-K6536). In contrast, the ectopterygoid of Late Carboniferous and Early Permian stem reptiles including araeoscelidians (e.g. *Araeoscelis* [157], figure 3) and acleistorhinids (e.g. *Delorhynchus* [120], figure 5) and bolosaurids (e.g. *Belebey* [61], figure 2) is rectilinear, lacking a posterolateral process and is poorly emarginated by the subtemporal fenestra.

#### 5.1.7. Loss of an ossified sphenethmoid

The sphenethmoid is an ossification of the interorbital cartilage that is present in most Palaeozoic amniotes. When present, the sphenethmoid rests upon the cultriform process of the parabasiphenoid and occasionally braces the skull roof dorsally. This condition is nearly ubiquitous in the earliest diverging synapsids (e.g. *Oedaleops* [49]) and stem reptiles (e.g. araeoscelidians [157]), but is also present in various ‘parareptile’ clades, including mesosaurids [89], acleistorhinids [11] and procolophonians [64]. An ossified sphenethmoid is absent in *Milleropsis* and other millerettids (e.g. *Milleretta*, BP/1/3822). The sphenethmoid is absent in neodiapsids (including Sauria, Rieppel and DeBraga [254]). Olson [255] described the sphenethmoid as present in *Youngina*, in which it is fused to the parabasiphenoid and flanks the basipterygoid processes of the parasphenoid. However, the sphenethmoid of early amniotes forms an interorbital septum—being present *anterior* to the posterior margin of the orbit—and does not represent an ossification of the presphenoid cartilage (*sensu* [255]). Further, scans of the *Youngina* specimens (BP/1/2871 and BP/1/70; [69]) reveal that a sphenethmoid ossification is not present in this genus, contra the observations of Olson [255] and consistent with its phylogenetic position as a neodiapsid.

#### 5.1.8. Loss of epipterygoid contribution to basicranial recess and implications for cranial kinesis

In *Milleropsis* and other millerettids, the basicranial recess is formed solely by the pterygoid (figure 13), with no contribution of the epipterygoid to this recess [25]. This morphology is also present in procolophonians among ankyramorphs (e.g. *Macroleter* [61]), *Orovenator* [15] and neodiapsids (personal observation *Youngina* BP/1/2871) including saurians (e.g. *Prolacerta* [56]). In these taxa, the epipterygoid bears a medial process that roofs the region of the basicranial articulation but does not contribute to the basicranial recess. The epipterygoid contributes to the basicranial articulation in all other stem or early amniotes in which this region can be examined, including captorhinids [43], synapsids [59], araeoscelidians [3], acleistorhinids [96] and bolosaurids [61]. Therefore movement along the basal articulation was limited in early stem reptiles. The loss of an epipterygoid contribution to the basicranial articulation in millerettids may be an important forerunner to the cranial kinesis exhibited in early saurians. The basicranial articulation of millerettids probably promoted more movement allowing the basicranial recess to be a more synovial joint compared with plesiomorphic taxa such as Captorhinidae. The medial lip of the epipterygoid in the millerettids and neodiapsids would have still severely limited anteroposterior movement along this joint, in contrast to squamates in which the epipterygoid is rod-like and located far posterior to the basal articulation [25,256,257].

#### 5.1.9. Abducens nerve (CN VI)

In *Milleropsis*, the foramen marking the pathway of the abducens nerve is visible as a foramen and groove that travels through the clinoid processes of the parabasisphenoid, just lateral to the prootic articulation (figure 19*c*). This morphology is also observable in neodiapsids (e.g. *Youngina capensis*, personal observation BP/1/2871) and is used as a character in several saurian matrices (e.g. [7,60]). In early amniotes, including traditional synapsids such as varanodontines (e.g. *Mycterosaurus*, personal observation FMNH UC 692) and the ophiacodontid *Ophiacodon* [59], as well as traditional ‘eureptiles’ including captorhinids (personal observation OMNH 44816 [43]) and araeoscelidians [4], the foramen marking CN VI pierces the dorsum sellae of the parabasisphenoid. The functional implications of this trait are unclear, and this trait may be at least partially linked to the dorsoventral development of the dorsum sellae.

### 5.2. ‘Aggregations’ in the fossil record or widespread burrow sharing in the Karoo

Aggregations and inferences of parental care based on fossil aggregations of large and small individuals are documented in a wide range of early reptiles and probable synapsids throughout the Palaeozoic. The earliest documented ‘social’ aggregation is that of the varanopid synapsid *Dendromaia umakiensis* [258] from the Late Carboniferous of Nova Scotia, in which a somatically mature individual is preserved with a possible conspecific individual in the same tree stump. In the middle Permian *Tapinocephalus* Assemblage Zone of the Karoo Basin, South Africa, the varanopid *Heleosaurus scholtzi* is known from an aggregation that includes five individuals, including a single large individual thought to represent an adult [61,73]. The probable synapsid identity of these taxa has had widespread implications in the evolution of mammalian sociality, with studies ascribing some level of mammalian-like sociality soon after the origins of amniotes [149,258].

Despite the emphasis placed on origins of mammalian sociality from aggregations of fossil synapsids [258,259], aggregations of undisputed stem reptiles outnumber aggregations of early synapsid occurrences in the well-sampled Karoo Basin of South Africa [73,260]. An aggregation of five conspecific individuals of a ‘younginiform’ was reported within a burrow structure from the *Endothiodon* Assemblage Zone (late Permian) of South Africa (SAM-PK-K7710 [260]), whereas the holotype of *Milleropsis pricei* (BP/1/720) consists of at least nine articulated individuals from a burrow structure from the middle *Cistecephalus* Assemblage Zone, with Individual II from this aggregation being up to 65% larger and somatically more mature than other conspecific individuals. In the late Permian and Early Triassic of South Africa, respectively, owenettid procolophonoids are known from aggregations: SAM-PK-K11289 (Lutendo Mukwevho, 2024, personal communication) with more than 30 individuals and *Saurodektes kitchingorum* with more than 12 individuals (JC, personal observation JC).

Interpretations of these stem reptile aggregations have differed markedly from those of probable synapsids (e.g. *Dendromaia* and *Heleosaurus* [73,258], despite their similar syndromes of preservation (i.e. aggregations of multiple individuals from different size classes). The presence of a large individual in specimens of these varanopid synapsids has yielded inferences of parental care in these taxa [149,258], with studies even suggesting the name ‘*Microvaranops parentis*’ for the *Heleosaurus* aggregation [241]. In contrast, stem reptile aggregations have been interpreted as evidence of juvenile burrow sharing to improve diurnal thermoregulation, aestivation and water-retention [260], although the *Milleropsis* aggregation (BP/1/720) includes a large individual preserved with several juveniles, similar to the mode of preservation of these varanopids.

We suggest that rather than proposing explanations for Palaeozoic amniote social aggregations that draw on more derived members of the respective clades (parental care for synapsids and thermoregulation for stem reptiles), workers should consider that aggregation syndromes of both early synapids and early reptiles may reflect similar behavioural patterns in both groups, and acknowledge that a wide range of alternative explanations are possible. Parental care in all these examples, for both synapsids and stem reptiles, is certainly possible, but at this stage largely equivocal. These instances could also be feasibly interpreted as seasonal aggregations, reproductive aggregations or communal nesting [261–263] until more robust evidence is provided. Although the causal mechanism of each of these aggregations is undoubtedly unknown, the occurrence of aggregations among early amniotes may be a result of a shared behavioural explanation, one that may be plesiomorphic for Amniota.

## 6. Conclusions

Our review of the cranial anatomy of *Milleropsis* based on high-resolution PPC-SRµCT imaging reveals previously unrealized anatomy for late Permian stem reptile and corrects widespread historical misrepresentations based on the poorly prepared holotype of this taxon. The skull of *Milleropsis* and, in particular, the occiput, palate and braincase bear numerous similarities with coeval neodiapsids; features that are absent in typical ‘eureptiles’ including the Captorhinidae and Araeoscelidia. We find at least eight cranial similarities between millerettids and neodiapsids lacking in other stem-reptiles that provide strong anatomical evidence that millerettids represent a more crownward lineage within Reptilia. In particular, the presence of a tympanic fossa, modification of the basicranial articulation, reduced stapes and patterns of cranial nerve innervation (e.g. pathway of the abducens nerve through clinoid processes) in *Milleropsis* are most similar to shared characteristics among neodiapsids, suggesting that sensory evolution and adaptations for cranial kinesis were underway well before the origin of the crown group, Sauria. Our work demonstrates the importance of PPC-SRµCT in revealing the neurocranial anatomy of small-bodied Palaeozoic stem reptiles and highlights the importance of anatomical studies on plesiomorphic representatives of extremely derived groups, such as the Millerettidae. These findings clearly emphasize the need for further scrutiny of classical phylogenetic hypotheses of Reptilia, including the supposed Parareptilia–Eureptelia dichotomy and the purported ghost lineages leading to Neodiapsida.

## Data Availability

Scans of *Milleropsis* specimen BP/1/720 are available on Morphosource (https://www.morphosource.org/concern/media/000125334?locale=en).
